# Kolmogorov compression complexity may differentiate different schools of Orthodox iconography

**DOI:** 10.1038/s41598-022-12826-w

**Published:** 2022-06-24

**Authors:** Daniel Peptenatu, Ion Andronache, Helmut Ahammer, Richard Taylor, Ioannis Liritzis, Marko Radulovic, Bogdan Ciobanu, Marin Burcea, Matjaz Perc, Tuan D. Pham, Bojan M. Tomić, Cosmin Iulian Cîrstea, Adrian Nicolae Lemeni, Andreea Karina Gruia, Alexandra Grecu, Marian Marin, Herbert Franz Jelinek

**Affiliations:** 1 Research Center for Integrated Analysis and Territorial Management, Faculty of Geography, University of Bucharest, 4-12 Regina Elisabeta Boulevard, 030018 Bucharest, Romania; 2 GSRC, Division of Biophysics, Medical University of Graz, 8010 Graz, Austria; 3 Physics Department, University of Oregon, Eugene, OR 97403 USA; 4 Key Research Institute of Yellow River Civilization and Sustainable Development and Collaborative Center On Yellow River Civilization, Laboratory of Yellow River Cultural Heritage, Henan University, Minglun Road 85, 475001 Kaifeng, Henan China; 5 Department of Experimental Oncology, Institute of Oncology and Radiology of Serbia, Pasterova 14, 11000 Belgrade, Serbia; 6 Mural Art Department, Faculty of Decorative Arts and Design, Bucharest National University of Arts, General Constantin Budisteanu 19, 010773 Bucharest, Romania; 7 Union of Visual Artists in Romania, Băiculești 29, 013193, Bucharest, Romania; 8 Faculty of Administration and Business, University of Bucharest, 4-12 Regina Elisabeta Boulevard, 030018 Bucharest, Romania; 9 Faculty of Natural Sciences and Mathematics, University of Maribor, Koroška cesta 160, 2000 Maribor, Slovenia; 10 Department of Medical Research, China Medical University Hospital, China Medical University, Taichung, 404332 Taiwan; 11 Alma Mater Europaea, Slovenska ulica 17, 2000 Maribor, Slovenia; 12 Complexity Science Hub Vienna, Josefstädterstraße 39, 1080 Vienna, Austria; 13 Center for Artificial Intelligence, Prince Mohammad Bin Fahd University, Khobar, 31952 Saudi Arabia; 14 Institute for Multidisciplinary Research, University of Belgrade, 1 Kneza Višeslava st., 11030 Belgrade, Serbia; 15 “Dumitru Stăniloae” Doctoral School, Faculty of Orthodox Theology, University of Bucharest, Sf. Ecaterina 2, 040155 Bucharest, Romania; 16 Department of Biomedical Engineering and Health Engineering Innovation Center, Khalifa University, 127788 Abu Dhabi, United Arab Emirates

**Keywords:** Applied mathematics, Mathematics and computing, Scientific data

## Abstract

The complexity in the styles of 1200 Byzantine icons painted between 13th and 16th from Greece, Russia and Romania was investigated through the Kolmogorov algorithmic information theory. The aim was to identify specific quantitative patterns which define the key characteristics of the three different painting schools. Our novel approach using the artificial surface images generated with Inverse FFT and the Midpoint Displacement (MD) algorithms, was validated by comparison of results with eight fractal and non-fractal indices. From the analyzes performed, normalized Kolmogorov compression complexity (KC) proved to be the best solution because it had the best complexity pattern differentiations, is not sensitive to the image size and the least affected by noise. We conclude that normalized KC methodology does offer capability to differentiate the icons within a School and amongst the three Schools.

## Introduction

An objective quantification of an artwork presents a challenge, and as such new objective methodological approaches become a necessity^1–3^. The characteristics of an artwork can be quantified and compared by considering characteristics such as shape, colors and brightness^4,5^.

The analysis of the complexity of Jackson Pollock’s artwork was approached by fractal analysis ^6^, indicating a series of similarities between Pollock's artistic expression and fractals^7^. These analyses have shown that Pollock is a unique artist of gestural expressionism due to his painting of complicated line patterns, which consistently comply with fractal scaling criteria. Fractal analysis was also used to differentiate paintings of individual artists, artistic directions and historical periods. Fractal dimension represents a direct measure of the complexity of an image^9,10^. Quantitative analyses of paintings from different historical periods have highlighted a number of characteristics of painting styles, such as image compression^11^, entropy^1^, and complexity^5^. A new trend based on compression has been introduced in statistical inference, learning theory and artificial intelligence^12^.

Leonardo da Vinci (1402–1519) used documentary geometry to create the first deformed grids, which when viewed from a certain angle appear normal. Examples can be seen in Illusion of the Square, and Study in Perpetual Motion^13^. Salvador Dali (1904–1989), a Spanish surrealist painter, used drawings with strong geometric-topological elements in his paintings. Dali depicted the four-dimensional space (e.g. hypercube^14^) in the two dimensional paintings in many of his works, as can be seen in The Last Supper, and The Cross. Van Gogh's Starry Night (1889) features chaotic vortices that accurately follow mathematical descriptions of turbulence in fluid materials, such as the turbulence of water in a river or a tornado. St Maurits Escher (1898–1972) is considered the father of this art direction. Escher is best known to crystallographers for his successful mosaic-dividing technique. He created images based on the laws of symmetry, set theory, perspective, topology and crystallography, as seen in Multiple view points and Impossible stairs: Relativity, (1953)^15,16^.

Artists often design their work in balance and symmetry. But there are fractal constructs or faults/damage in the structure of the paintings if we look at some paintings in detail: deposits, bloating, losses, different overpainted levels, fragments-palimpsest, and more^17^.

The introduction of information technology helps in, (a) the preservation and promotion of cultural heritage by digitization, and, (b) attribution of a work of art to a named painter or School of Art by digital image analysis. The preservation and promotion of cultural heritage began with the need to capture works of art and monuments with visual media to make them accessible to the general public without being damaged at exhibitions. Attributing a work of art to a named painter is an important aspect of art history and was initiated in the history of art and authenticity testing for characterization and provenance purposes as well as studies of a painter’s style. At any rate, in all the above examples it seems that beauty in art, which exists in complexity and fractals, may have consciously or subconsciously inspired such great painters. Two paintings by Piet Mondrian were analyzed by Bountis et al.^18^ who computed the fractal dimension by using the box counting approach and linear regression. This analysis suggested that his depiction of tree foliage exhibits fractal patterns of a specific dimension.

The methodology proposed here identifies the characteristics of each painting school in a very simple way. Based on the above, the question is whether there are differences between the schools of Byzantine Church painting styles and how they may be quantified. To gain a better understanding of those differences in painting styles between the three epochs, a sensitive and accurate fractal analysis method first needs to be identified. Artificially generated fractal surface (FS) images were used to validate the ability of the Kolmogorov Complexity (KC) algorithm to differentiate between different images. We aimed to differentiate 8-bit grayscale images, 16-bit grayscale images, and color images by using the Inverse Fast Fourier Transform (FFT) and the Midpoint Displacement (MD) algorithms^19^ with the KC^20^.

The aim of the present study is to analyze the three schools of iconography by investigating 1200 icons in 3 sets of 400 icons, belonging to three Christian-Orthodox countries: Greece/Byzantine Empire, from the XIII–XIV centuries, Russia from the XIV–XV centuries and the Romanian Countries from the XV–XVI centuries.

## Results

### Comparison between Kolmogorov Complexity (KC) analysis by its absolute values and normalized values

**Figure 1:**
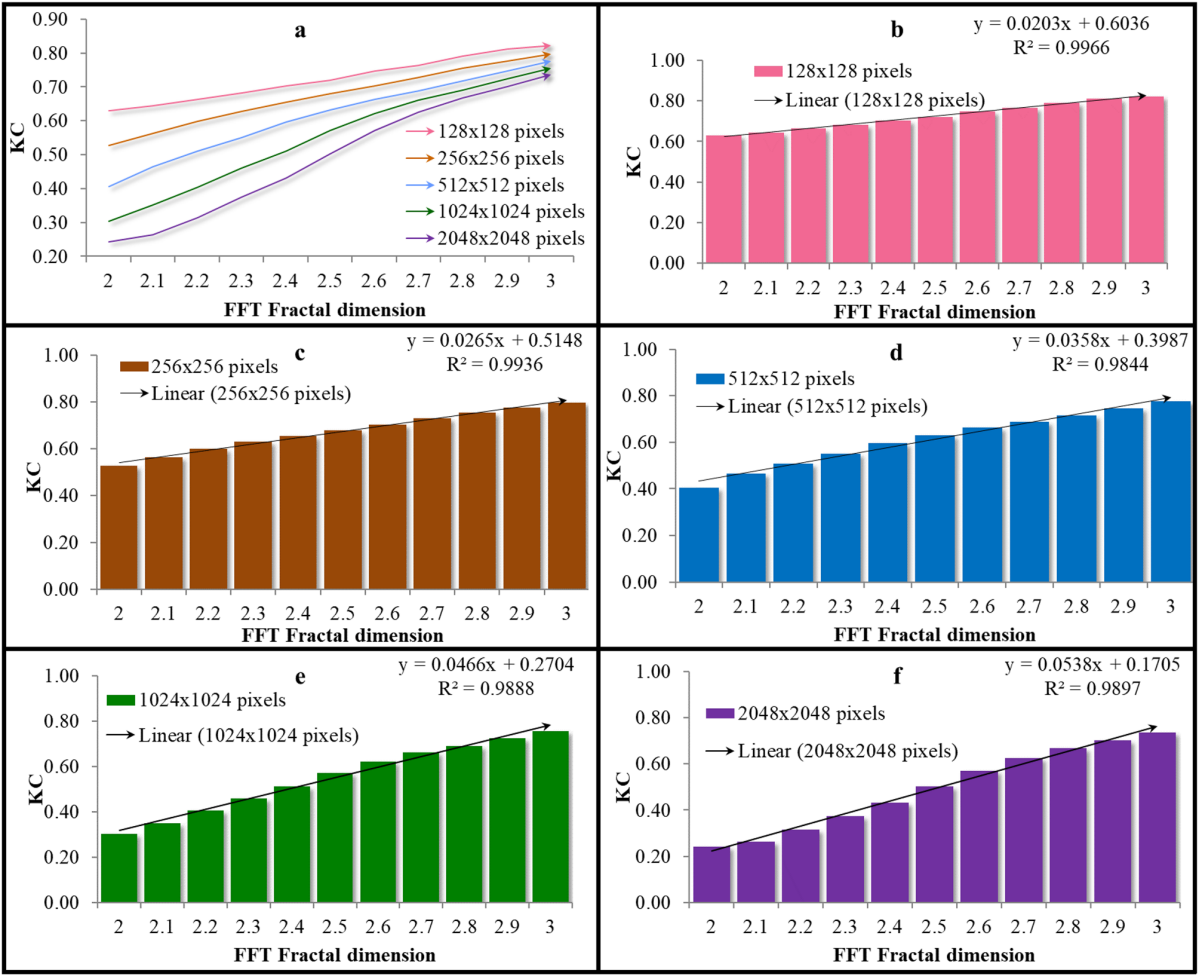
Testing the Kolmogorov Complexity (KC) analysis on automatically generated fractal surface images using the Inverse FFT algorithm. Each bar corresponds to averages for 20 generated images. (**a**) Absolute KC values without normalization, depending on the image resolution; (**b**) Normalized KC values, depending on the resolution of the images; (**c**) Normalized KC values for images with different fractal dimensions and a resolution of 256 × 256, (**d**) resolution of 512 × 512, (**e**) 1024 × 1024, (**f**) 2048 × 2048. Without KC normalization, images with different resolutions cannot be compared by KC (**a**), while KC normalization allows the comparison (**b**). At different resolutions (**c**–**f**), when images have the same FD (between 2.0 and 3.0) although the results are not similar, they are still comparable. It turns out that KC normalization allows differentiation of images even when they differ in size and resolution.

Analyzed images do not have the same resolution because of the different size of the paintings. For this reason, as KC depends on the image size, its actual values of are not informative. Thus, we used absolute and normalized values of to compare KC using 11 sets of 8-bit grayscale images. Figure 1 highlights the need for image normalization, whereby1a and 1b show that without normalization, KC is dependent on the image resolution. An image size of 1024 × 1024 yielded 200–300% larger values compared to the 512 × 512 size, although the content and complexity of the images were the same. In contrast, the normalized KC values differed only by 0–30%. Furthermore, KC normalization also ensured that values were between 0 and 1, and that differences deriving from image resolution are small (Fig. 1c–f).

**Figure 2:**
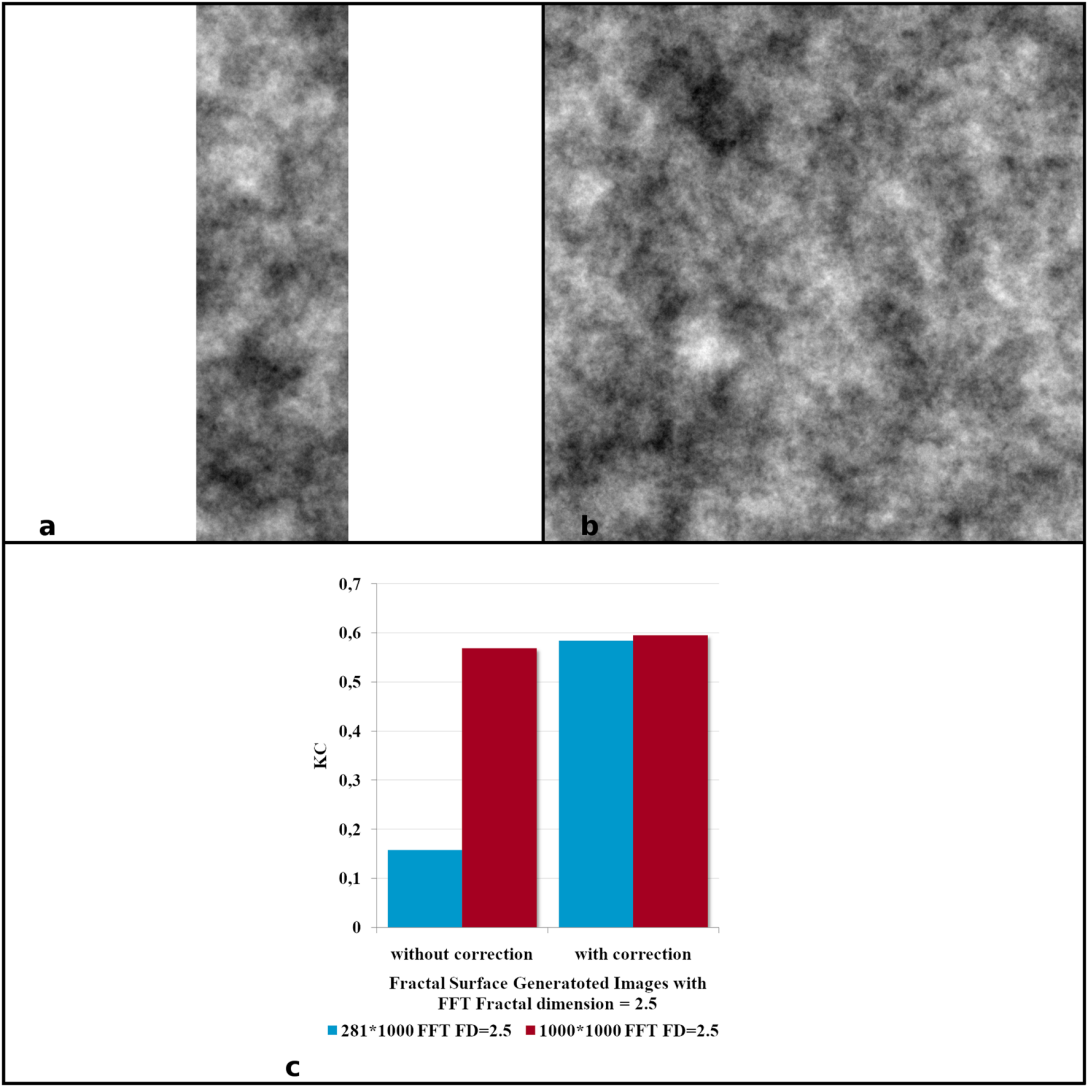
FS images in two resolutions and KC fractal; (**a**) FS images generated with Inverse FTT algorithm and fractal dimension of 2.5 and the lowest resolution of an analyzed icon (281 × 1000) and (**b**) the highest resolution of an analyzed icon (1000 × 1000). (**c**) Each bar corresponds to a single generated image. Although generated images under (**a**,**b**) have the same fractal dimension, their KC differs greatly due to the difference in size of the two images. Normalization of KC resolves this problem as both images having similar KCs (**b**).

For further validation, we generated two images with the Inverse FFT algorithm and fractal dimension of 2.5 and lowest (281 × 1000 pixels) (Fig. 2a) and the highest resolution (1000 × 1000 pixels) resolution (Fig. 2b) of the icons in our analysis. Figure 2c shows the usefulness of this correction, as well as the ability of KC to eliminate the bias imposed by different resolutions of the images subjected to fractal analysis. These results based on FS images and KC suggested that KC was an appropriate method to analyze the three sets of images with Byzantine icons of the three schools of church paintings. After normalization, the results were comparable for all the 1200 icons.

It is therefore proposed that without normalization KC would present very large differences between resolutions, even exceeding 200–300%. With the normalization there are still differences in resolution, but our images do not differ much in resolution. Thus, as can be seen in Fig. 2, KC presents very close values for images at different resolution 281 × 1000 versus 1000 × 1000 pixels which makes us consider that for our sets of icons KC can be suitable in quantifying visual complexity.

### Kolmogorov Complexity analysis of FS images in 16-bit and RGB grayscale at different resolutions

**Figure 3:**
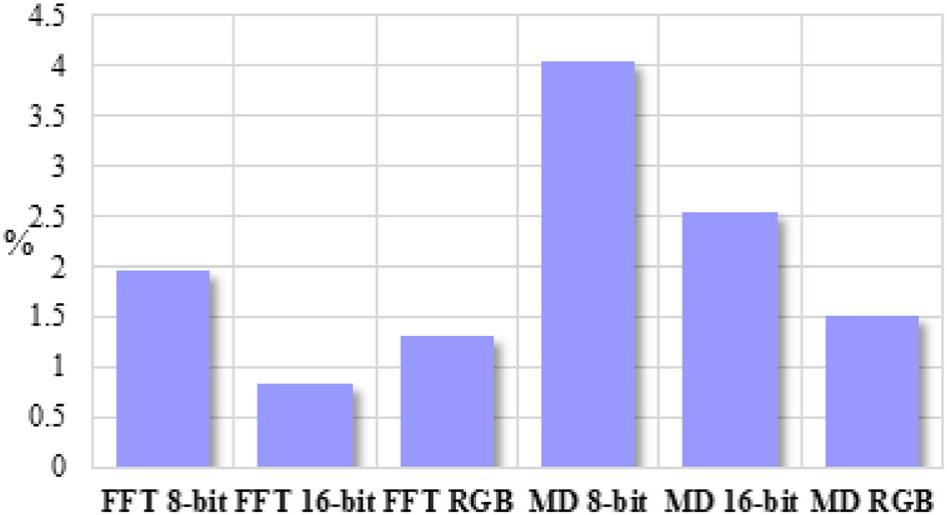
The differences of the ratio between Kolmogorov Complexities for images generated with the Inverse FFT or MD algorithm and Fractal dimension was 2.5 with 8-bit, 16-bit, Red–Green–Blue (RGB) image formats and the lowest resolution (281 × 1000 pixels) and the largest icon (1000 × 1000 pixels). Each bar indicates a ratio between KC and resolution. In all the analyzed situations normalized KC for the smallest and largest analyzed images differed by only 2% in the situation of images generated with Inverse FFT algorithm and up to 4% for images generated with MD algorithm.

Figure 3 shows that KC can be used for analysis of inverse FFT and MD generated images, as well as 16-bit and RGB grayscale images, where other fractal or entropy approaches have low relevance (see the supplementary material).

Concerning the methodology of using artificially generated FS images it may appear that artificially generated FS images have no structure or pattern visually close to the paintings, in lieu of the generated images which show visual complexity that increases from those generated with algorithm with FD = 2.0 to 3.0. In fact, these artificial images have structure / pattern visually close to the paintings and are compatible to the paintings and with fractal dimensions close to the paintings. The effectiveness of our methodology has been tested on three sets of Jackson Pollock’s painting. The classical fractal dimension (box counting fractal dimension, BCFD) of Jackson Pollock’s painting used by Alvarez-Ramirez et al.^21^ were compared to our KC results. Indeed, the results obtained with KC are extremely satisfactory and confirm those obtained by the classical approach and prove that KC can quantify visual complexity (see Supplementary material).

### Normalized KC analysis of the three sets of icons

**Figure 4:**
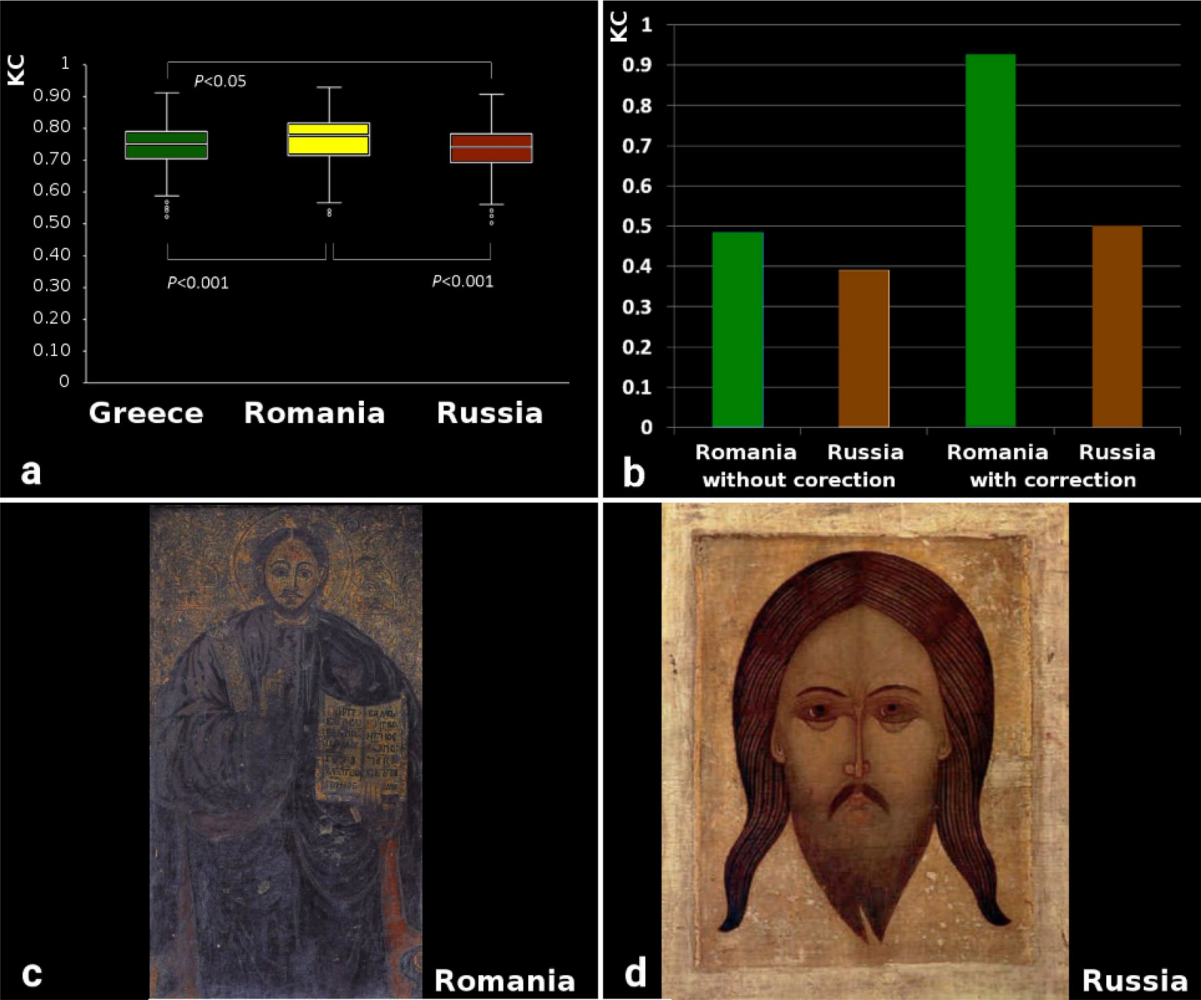
(**a**) KC values for the three schools of Byzantine iconographic painting (Greek, Romanian, and Russian school). Each bar indicates an average of 400 images with standard errors of the mean. The bootstrap-corrected *P* values obtained by the independent samples t-test are indicated. (**b**) KC values without and with normalization (our proposed correction), (**c**) an icon with 547 × 1000 pixels resolution, belonging to the Romanian school; (**d**) an icon with a resolution of 817 × 1000 pixels, belonging to the Russian school. (**b**) shows the usefulness of KC normalization, because without normalization (**c**) and (**d**) have close KC values, although (**c**) is much more complex.

Figure 4a shows the average KC values for each of the three groups of 400 icons. Romanian icons showed the highest complexity, due to the increased saturation of colors, and the strongest contrast, with more shades of colors. KC values without and with normalization for Romanian and Russian schools are shown in Fig. 4b. Even if the compositions of the Romanian icons were simpler in their geometric structure, stylized, reduced to the essential, but with many symbols taken from tradition, which determined higher complexity (Fig. 4c). In contrast, Russian iconographers emphasized complex geometric structures and used sober colors, which prompted interiorization; this is due to the strong emphasis on the practice of hesychasm. Due to the approach towards contemplation and asceticism, the Russian iconographers formed in this sense a special chromatic modality with all the colors being grayed, and transparent backgrounds from successive washings and brush vibrations. For these reasons, the KC of the Russian icons is lower than that of the Romanian ones (Fig. 4d). Figure 4b shows the difference in KC, with and without normalization, between the two extreme icons. It is observed that without normalization the difference is smaller.

The intermediate values of KC for Greek icons are possibly a consequence of their technique which involved mixing all colors with a little ocher, yellow and gold, while matte backgrounds were repeated at least two times. Techniques with tinted washers were also used, arranged in light areas or shades in addition to classic lighting (Fig. 4a). To see the effects of KC normalization in image analysis, we present results with and without normalization the two icons with a resolution of 547 × 1000, belonging to the Romanian school (Fig. 4c) and with a resolution of 817 × 1000, belonging to the Russian school (Fig. 4d).

Noticeable is the small changes in mean KC values and their significant contrasting the three groups is worth of attention. The variations occur in the second decimal because all 1200 icons have KC between 0.5 and 1 and normalized KC has values only between 0 (monochromatic image) and 1 (pure noise).

The averages of the icons of the 3 schools of Ortodox iconography are close: Romania 0.764, Greece 0.743 and Russia 0.732. The main cause is the heterogeneity of the themes of the icons. For example, there are 19 icons with Jesus Christ for the Greek school, 14 for the Romanian school and only 7 for the Russian school. For the Crucifixion of Jesus, 5 icons belong to the Romanian school, 9 to the Greek school and 7 to the Russian school. For the Assumption of the Virgin Mary, 8 icons belong to the Romanian school, 7 to the Greek school and 4 to the Russian school. For Virgin Mary and Jessus Christ 46 icons belong to the Romanian school, 21 to the Greek school and 17 to the Russian school. We consider that the problem is not from the methodology but from the thematic heterogeneity of the icons of the 3 sets. For these reasons, we analyzed the 4 case studies in the next step.

**Figure 5:**
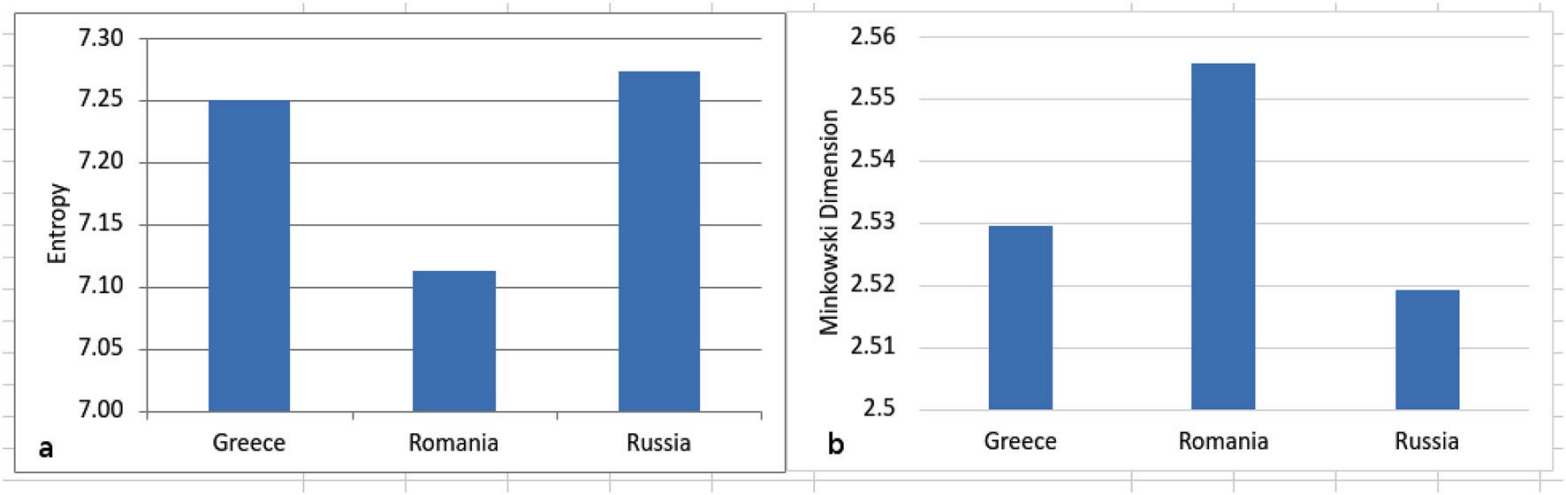
Mean values for the Entropy (**a**) and Minkowski Dimension (**b**) analysis of the three painting schools.

**Figure 6:**
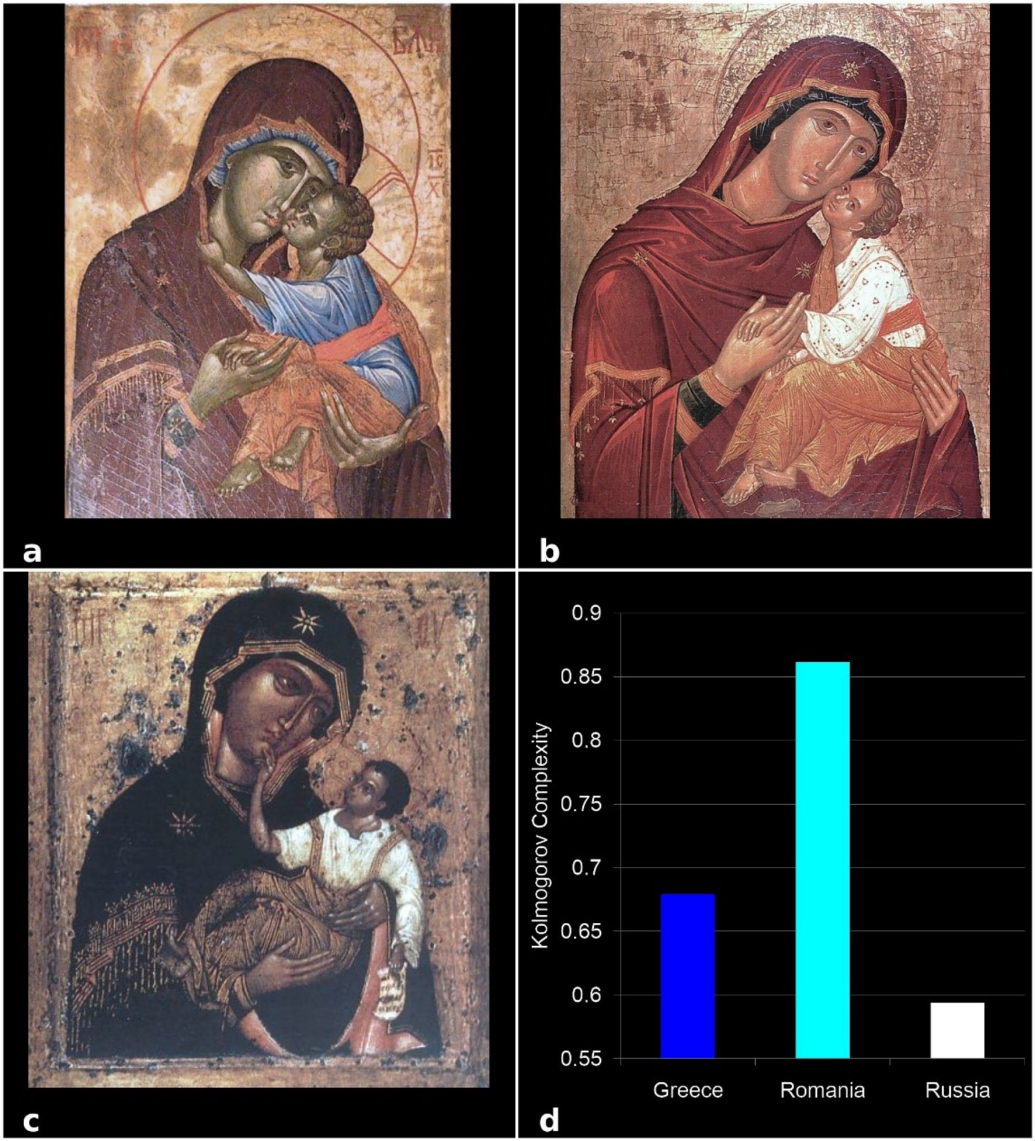
KC analysis of the Virgin Mary and Jesus Christ icons as single examples each from the three schools of Byzantine iconographic painting: (**a**) Greek, (**b**) Romanian, and (**c**) Russian school, (**d**) KC values. Each bar indicates a KC value of the single icon.

**Figure 7:**
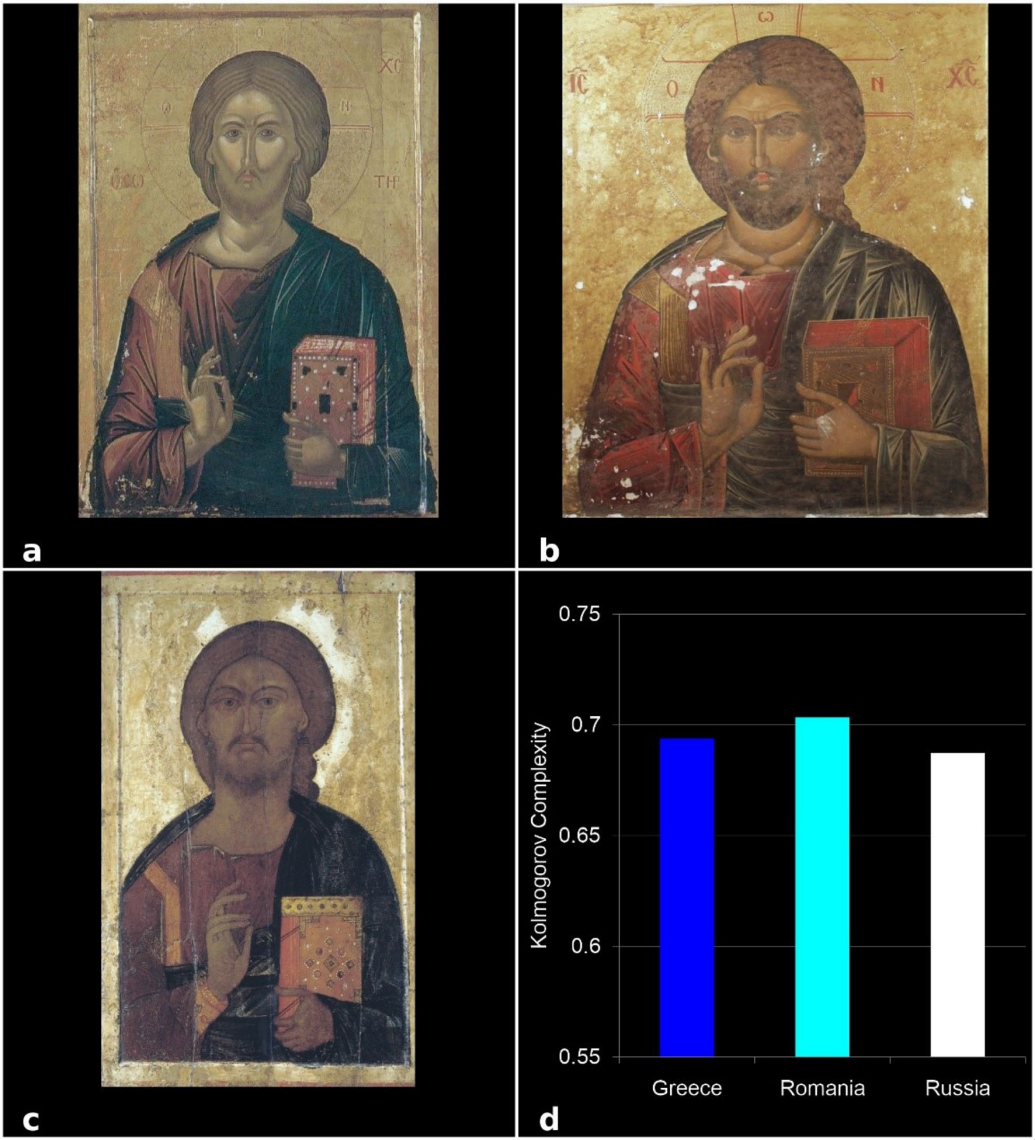
KC analysis of the Jesus Christ icons from the three schools of Byzantine iconographic paintings: (**a**) Greek, (**b**) Romanian, and (**c**) Russian school, (**d**) KC values. Each bar indicates a KC value of the single icon.

**Figure 8:**
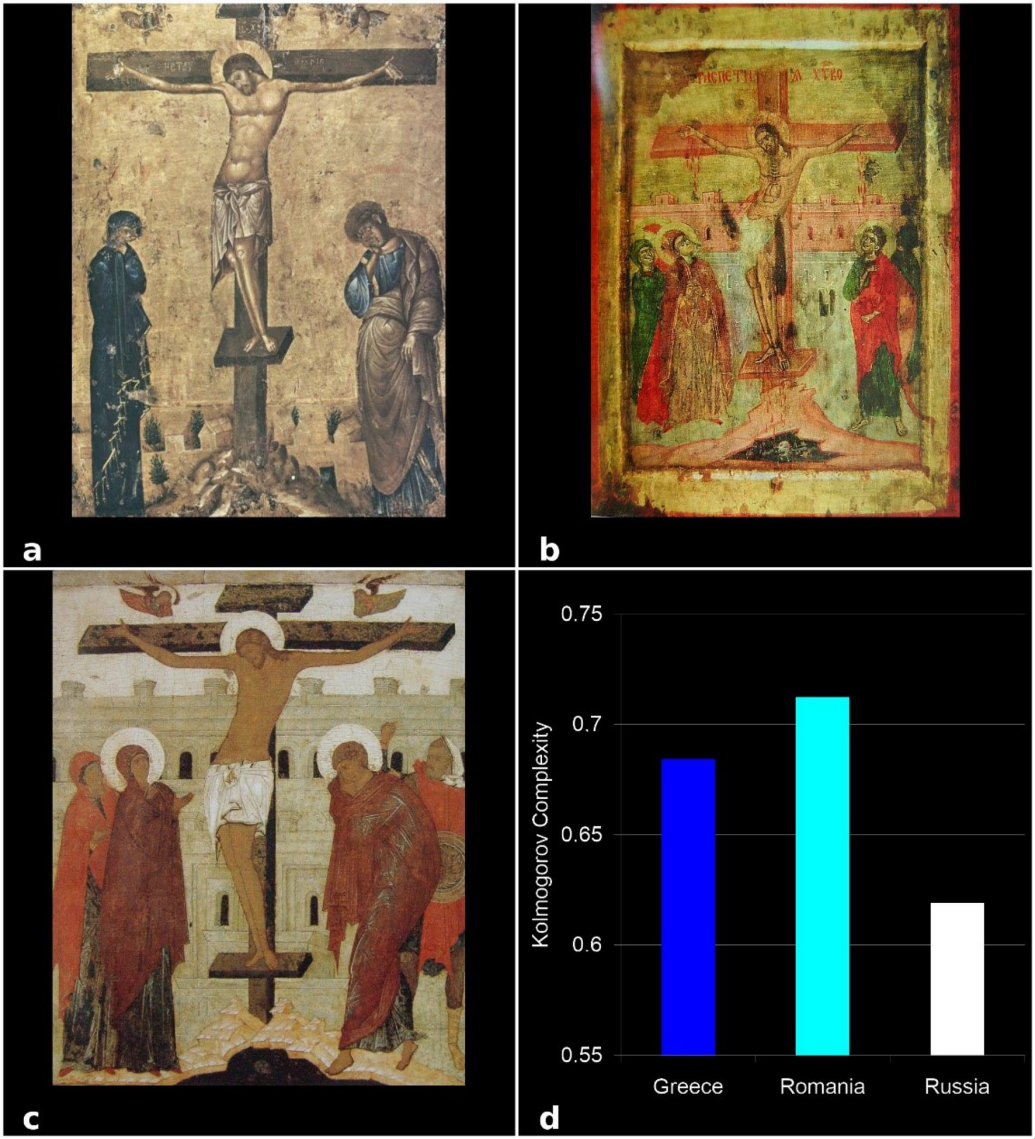
KC analysis of The Crucifixion of Jesus icons from the three schools of Byzantine iconographic painting: (**a**) Greek, (**b**) Romanian, and (**c**) Russian school, (**d**) KC values. Each bar indicates a KC value of the single icon, as indicated.

The existence of small differences between the 3 groups analyzed is due to the very high heterogeneity of the biblical scenes described or of the saints painted in icons. Thus, the median of the Romanian icon set is 0.78, 0.75 for the Greek icons and 0.74 for the Russian icons. For mean 0.76, 0.74 and 0.73 respectively. Being heterogeneous sets, the difference between the minimum and maximum values is large for all 3 schools. The Romanian icons have KC between a minimum of 0.53 and a maximum of 0.93; the Greek ones between 0.52 and 0.91 and the Russian ones between 0.50 and 0.90. SD has relatively low values, being 0.076 for the Romanian school, 0.072 for the Greek school and 0.074 for the Russian school. The proposed method can be applied when there are no major differences in resolution and when the theme of the icons is similar. Different themes will automatically give you different visual complexity. Therefore, in order to be able to differentiate the visual complexity of the 3 styles of Orthodox iconography, it was necessary to analyze the 4 case studies referred to different painting scenes (see Figs. 5, 6, 7, 8).

For validation we calculated as alternative methods Entropy and Minkowski Dimension for all 1200 icons (Fig. 5a,b). Minkowski results correlate very well with the normalized Kolmogorov Complexity, R^2^ being 0.9 for the Romanian school and 0.86 for the Greek and Russian Orthodox iconography schools (not shown). The Entropy analysis on the 3 sets of icons confirms the results of the analyzes with KC, the average values for the 3 schools being: Greek school 7.25, Romanian school 7.11 and Russian school 7.27. As in the case of FSG image analysis, the entropy of the images are an inverse to  KC  results.

### KC analysis of case studies

**Figure 9:**
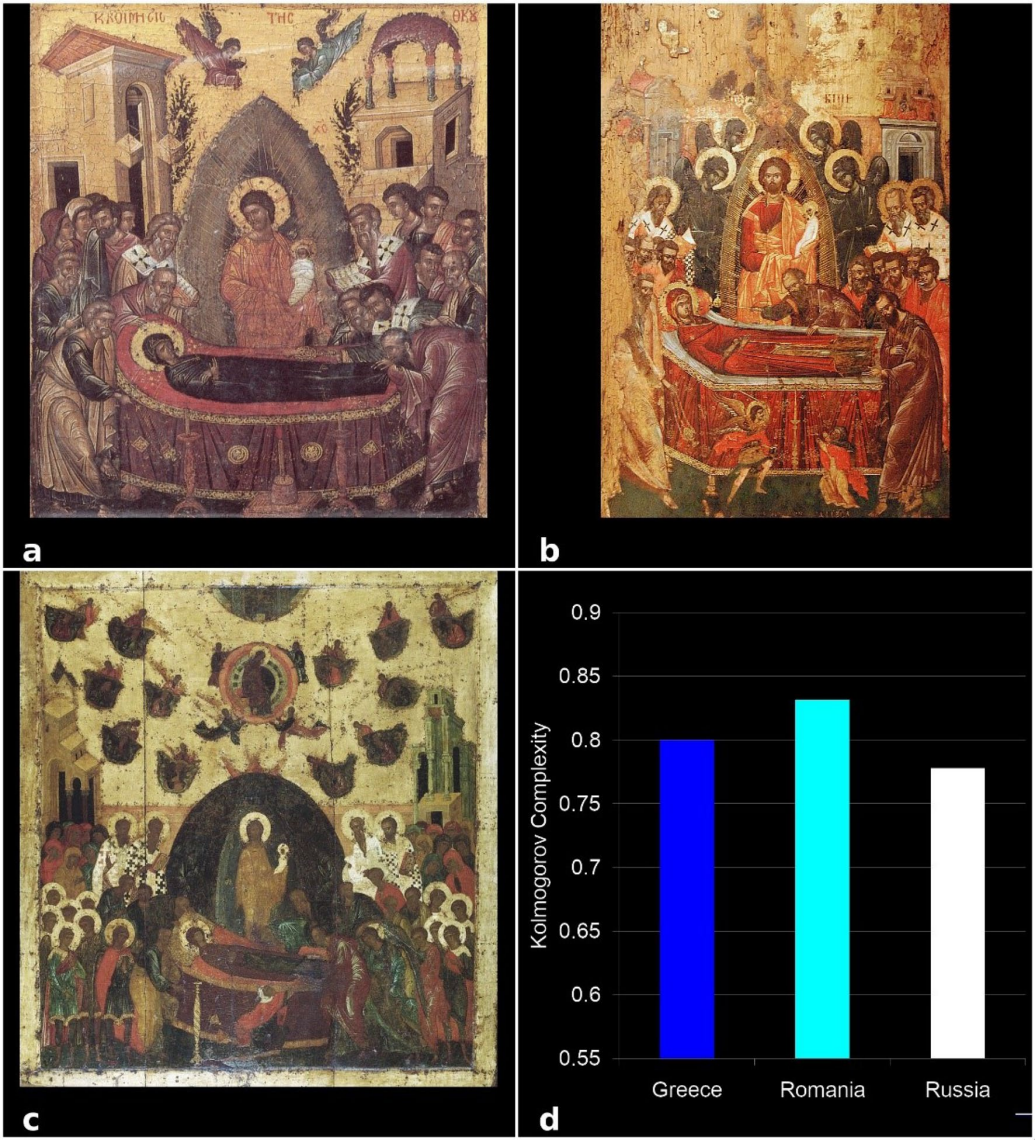
KC analysis of the Assumption of the Virgin Mary icons from the three schools of Byzantine iconographic painting: (**a**) Greek, (**b**) Romanian, and (**c**) Russian school, (**d**) KC values. Each bar indicates a KC value of the single icon, as indicated.

To validate these results, we analyzed 4 types of icons—the representative images from the three schools: Virgin Mary and Jesus Christ (Glicophilousa) (Fig. 6a–c), Jesus Christ Pantocrator (Fig. 7a–c), The Crucifixion of Jesus (Fig. 8a–c), and Assumption of the Virgin Mary (Fig. 9a–c). The analysis has shown that the style differences between the three schools were confirmed at the level of specific composition, the most complex being the Romanian ones, followed by the Greek and Russian ones. The icon of The Crucifixion of Jesus best displays the graphic, chromatic and technical differences presented previously (Fig. 8d), but these are also noticeable in the other three iconographic compositions (Fig. 6d, 7d, 9d); in those Figures the fractal correspond to only one icon per school. Our results indicate that the Romanian icons clearly highlight the particularities of chromatic complexity, which is computationally quantifiable and also visible to the naked eye.

## Discussion

Earlier investigations of making use of KC in various areas have produced interesting results. Our methodology and results extend this statistics to further directions and assessments focused on painted icons, with a brief discussion and correlation to earlier works. Here we have introduced the normalized Kolmogorov Complexity as the classification tool of Byzantine icons from the three schools of religious painting: Greek, Russian and Romanian. In the first stage, entropy and nine fractal parameters were tested: Logical depth (LD), Differential Box-Counting (Db_DBC), Relative Differential Box-Counting (Db_RDBC), Pyramid Dimension Gradient—PGM method (Dp_Gradient PGM), Minkowski Dimension—Blanket method (Dm_Blanket), Fractal FFT Dimension using Discrete Fourier Transformation (Df), Higuchi Dimension 1D (Dh), Higuchi 2D—KfoldDiff method (Dh-KfoldDiff) and Kolmogorov Complexity (KC) (see Supplementary material). The analyses were performed on 1100 grayscale images generated by the Midpoint Displacement (MD) and Inverse Fast Fourier Transformation (FFT) algorithms, at resolutions ranging from 128 × 128 to 2048 × 2048 pixels. Twenty images were generated for MD and FFT, respectively, for 11 classes of fractal dimension set to: 2.0 (minimum complexity), 2.1, 2.2, …, 3.0 (maximum complexity).

It was necessary to perform the size-normalization of the images because the analyzed images differed in size and shape. All fractal and entropy analyses showed sensitivity to image size. Large differences in Kolmogorov Complexity between images with different resolution were resolved by normalization. Kolmogorov's complexity had the smallest differences in fractal dimension among the 20 images in the 11 classes analyzed and was also the least sensitive to the effects of salt and pepper and noise alteration.

Gruia et al.^22^ used Kolmogorov Complexity to investigate the spatial behavior of the dynamics of the economy in the Development Region of Bucharest-Ilfov in Romania. Thereby, they highlighted models of spatial dynamics of colored maps that helped to understand the sustainable economic development of regions. Their study demonstrated the usefulness of this method for exceeding the current methodological limits in analyzing the dynamics of territorial reality. Their color gradient maps were turned into grey tones of the number of employees and the turnover in five classes. In Gruia et al.^22^ the analyzed images were the same size and the 5 revenue classes for creative economies, created after the division of turnover values (in Ron), had the same chroma for each class with no chromatic alteration. In our study, we analyzed 1100 artificially generated images but also 1200 images of Byzantine icons with differences in size and degree of degradation due to their age. While Gruia et al., analyzed only dynamics of financial turnovers, in the current study we show that the Kolmogorov complexity is also useful in identifying patterns.

The use of fractal analysis in analysis of paintings is not new. There have been several fractal and non-fractal approaches. Milanović and Tomić ^10^ examined the existence of fractal patterns in Byzantine iconography. They proved that fractality is manifested in descending mode (apparent information, corresponding to the content of icon) and ascending mode (hidden causal information, corresponding to the self-organizing spatiality). Our study confirmed that Byzantine icons can be analyzed by fractal analysis and has revealed that, for a set of icons depicting the same scene, different complexities can be identified between the three analyzed schools.

Forsythe et al.^11^ used fractal analysis to explain the beauty perceived by an observer by measuring the visual complexity between natural and abstract images. These authors used GIF compression to approximate visual complexity and by combining it with the fractal dimension, they were able to explain the variability of judgments of the beauty perceived in art. They also indicated that if color is removed from the painting, viewers were unable to make meaningful judgments about its beauty. In comparison, we showed that KC indicates greater complexity of the Romanian Byzantine compared to the Russian and Greek icons possibly based on the more intense chromatic contrasts and partly possibly the higher degree of preservation as Russian and Greek icons are 1–2 centuries older.

Kim et al.^5^ investigated the three quantitative measures of images: brightness, use of individual colors, as well as color variety for the ten historical periods of art. They found a difference in color use, consistent with historical circumstances and a reduced variety of colors in the medieval period. They also showed that physical degradation through oxidation and corrosion suffered can generate bias in the analysis of old paintings. Based on this observation, we tested KC on artificially generated images with FFT and MD algorithms, to which degradation filters such as Gaussian noise and salt and pepper noise were applied. From the analysis of original and altered images, we noticed that KC exhibits approximately similar values, indicating that degradation from age produced negligible bias in the analysis of Byzantine icons.

Bratitsi et al.^23^ classified ancient ceramic shreds of unknown origin by analysis of their color indices on the chromatic scale (RGB). Though based on only RGB the differentiation of objects from color provides a general clue but includes uncertainties; hence a more sophisticated classification and attribution method is needed. Our study proves the satisfactory test regarding KC to quantifying patterns for 16-bit grayscale or RGB color images, and moreover compared the complexity of the artwork and managed to differentiate from which school of Byzantine painting an icon originated, with the provision that the icon indicates the same biblical moment.

Mureika et al.^24^ in 2005 used multifractal analysis to study the abstract expressionist artwork by different artists and showed that the blobs method can distinguish paintings belonging to different artists from the same artistic school. Also, Murieka et al.^2^ in 2010 used multifractal analysis to show that differences in fractal characters of paintings can be identified by examining indicators of the artist's physiology. We set out to find a technique for identifying an iconographic school based on the style differences approached and Kolmogorov Complexity t, for at least the four case studies, grouped the icons from the three schools.

Taylor et al.^7^ showed that Pollock refined over time his dripping technique by using fractal box-counting analysis. An increase in fractal dimension was thereby identified from 1.0 for paintings in 1943 when he used a single coat of paint, to 1.72 for paintings from 1952 for paintings with multiple coats. In 2002 Taylor et al.^8^ showed that Pollock succeeded in creating complex fractal patterns through this technique. Oancea and Rapa^9^ performed a series of fractal analyses on Pollock's paintings using box-counting, and identified the complexity of his works of art. In our current study, we used the normalization of the Kolmogorov Complexity because the icons had different degrees of degradation and different sizes. We showed that for the same types of Byzantine icons the complexity increased from the oldest Greek school to the Romanian school, the newest of the three schools analyzed. The higher complexity values of the Romanian Byzantine icons may be due not only to the more pronounced chromatic contrast, but also to the improvement of techniques or the adoption of some elements of detail from the other two pre-existing Byzantine painting schools.

Sigaki et al.^1^ analyzed 140,000 paintings made during a period of 1 millennium in art history by analyzing the entropy of permutation and statistical complexity. They showed that different artistic styles present different values of entropy and complexity, allowing hierarchical organization and grouping by styles. Shamir^3^ analyzed Pollock's original paintings obtained by his revolutionary technique of dripping paint on the horizontal canvas with other painting styles used by various painters who tried to imitate his style. This analysis showed that in 93% of cases, they could identify the Pollock's artworks. De la Calleja and Zenit^4^ analyzed the complexity of abstract paintings by calculating the Betti number and demonstrated that Pollock's paintings are superior to the abstract paintings of other artists who imitated his style. In our study we only aimed to analyze the Byzantine icons from the three schools and not the differences between the  painters. Subsequent studies will show whether the Kolmogorov Complexity can distinguish between painters.

Regarding the use of human perceptual measures such as visual complexity, cognitive complexity or others, instead of KC, as well as, the link between 'visual/perceptual/qualitative' feel of the paintings and the quantitative nature of these measures, this has been of concern. In fact, a multifractal analysis is questionable, because it would assume more than one range of scaling. Digital images have only a limited range of scales (3 may be 4), so the linear part in a double log plot can only be searched for these 3 to 4 decades (essentially only 3 to 4 points in the double log plot). Dividing this limited range into even smaller ones does not make sense and is meaningless. In the present work, the deviation from the linear part does not show the multi fractality, but the deviation from mono fractality, especially for paintings with a lot of non-fractal shapes. At any rate, there could always be a better parameter and the list of possible measures is quite long. In the present endeavor the best parameter, that of KC turned out to be a promising and good parameter for this study.

Even after normalization, the KC values vary from ~ 0.2 to ~ 0.65 (Fig. 1f) and referring to Fig. 4a, the differences between the mean KC values for the three groups of paintings (Greek, Romanian, Russian) seem low. The main cause is the heterogeneity of the themes (scenes) of the icons. These close KC values is not an undesirable result caused by the methodology but due to the thematic heterogeneity of the icons of the 3 sets. For these reasons, we analyzed the 4 case studies regarding different scenes.

That the Kolmogorov complexity had the smallest differences in fractal dimension among the 20 images in the 11 classes analyzed and was also the least sensitive to the effects of salt and pepper and noise alteration, no mathematical explanation can be given for this. However, the mathematical connection between Kolmogorov complexity and fractal dimension is not simple. Both can be interpreted as parameters that measure the content of complexity in an image, but both parameters do this in a different mathematical way.

## Conclusion

The presented fractal analysis is a useful tool in differentiating iconographic schools, each painting school having its own theological vision and emerging mathematical concept^25^. The KC normalization of the FS generated images showed that the increase in complexity was directly proportional to the increased fractal dimension. This holds true for different image resolutions. In this way, databases can be created with archetypal patterns that can quickly and accurately identify the unknown origin to the school and individual artist for paintings owned by institutions such as auction houses, private collections, and art museums.

From a practical point of view, this technical approach has an indisputable utility in recognition of some schools, starting from certain models and calculation of indicators that measure complexity. It should be emphasized that the method proposed in the present study does not claim to exhaust the mystery of the contemplation of the icon by methods related to technical rationality. Especially in the context of today's information society, with an overly technical mentality, it is important to recognize the spiritual view of reality presented by the icons. Obviously, this perspective does not cancel the effort of an analytical rationality, but it is fundamental to understand that spiritual feelings transmitted by the icons cannot be seen and acquired through algorithms.

KC can be a fast and useful tool in differentiating complexity and can be useful not only in iconography or in the analysis of works of art but also in classifying patterns in various fields such as medicine, biology, landscape ecology, remote sensing, geology, economic data spatialization^26–31^.

The described KC analysis and normalization could be used to investigate geographical routes followed by some iconic painters, which might be a valuable information on the cultural influences throughout history. The method proposed here might be applied in various fields based on its advantage of comparing images at different resolutions.

## Methods

Entropy and nine fractal parameters were employed in the exploration of the 1200 byzantine icon images, as well as artificially generated fractal surface (FS) images, to validate our methodology.

### Generating test images

**Figure 10:**
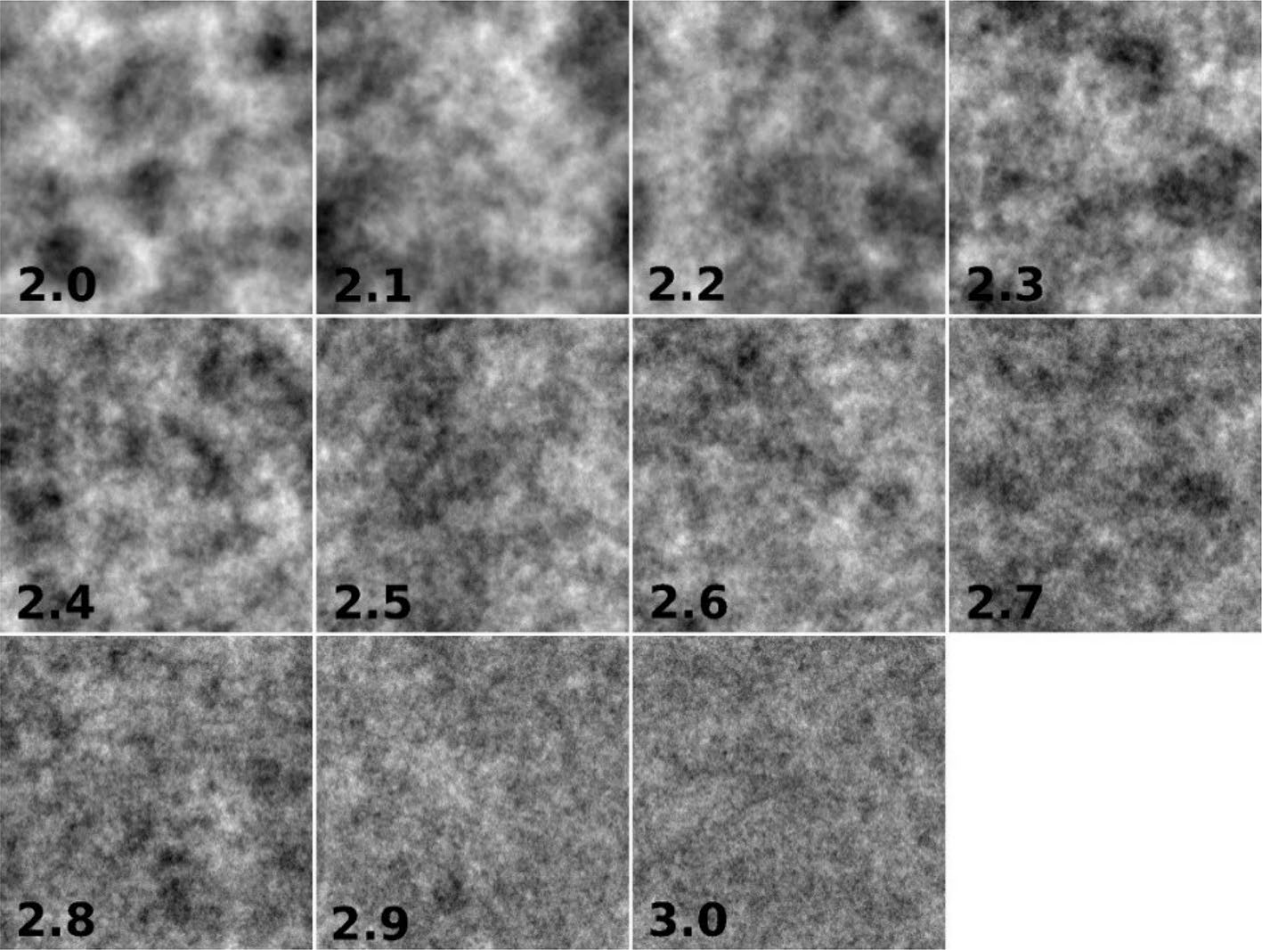
FS images of 8-bit grayscale applying the FFT algorithm and fractal dimension between 2.0 and 3.0.

To validate the proposed approach, we generated 5 × 11 sets of generated images with fractal surfaces using an Inverse FFT algorithm and fractal dimensions between 2.0 and 3.0. The grey scale values of a digital image represent a surface, a topological two-dimensional object that more or less fills the third dimension. Thus, the fractal dimensions of gray-scale images are in the range of two (minimum complexity) and three (maximum complexity). The generated test images were therefore also generated in this range. In any way, the painting on canvas is made by pigments and a paintbrush, thus produce an oftentimes perceptible relief. This together with the geometrical image provide 2 < FD < 3. Yet in a 2D image the FD can be even < 1; but since in our case we allow unlimited resolution, and everything is made of pixels, which have fractal dimension 2, the chosen 2 < FD < 3 is justified. Image resolutions were: 128 × 128, 256 × 256, 512 × 512, 1024 × 1024, and 2048 × 2048. Finally, each set contained 20 images. All images were generated in 8-bit grayscale, to compare the results of the proposed method with other fractal or non-fractal approaches (Fig. 10) (see the Supplementary material).

**Figure 11:**
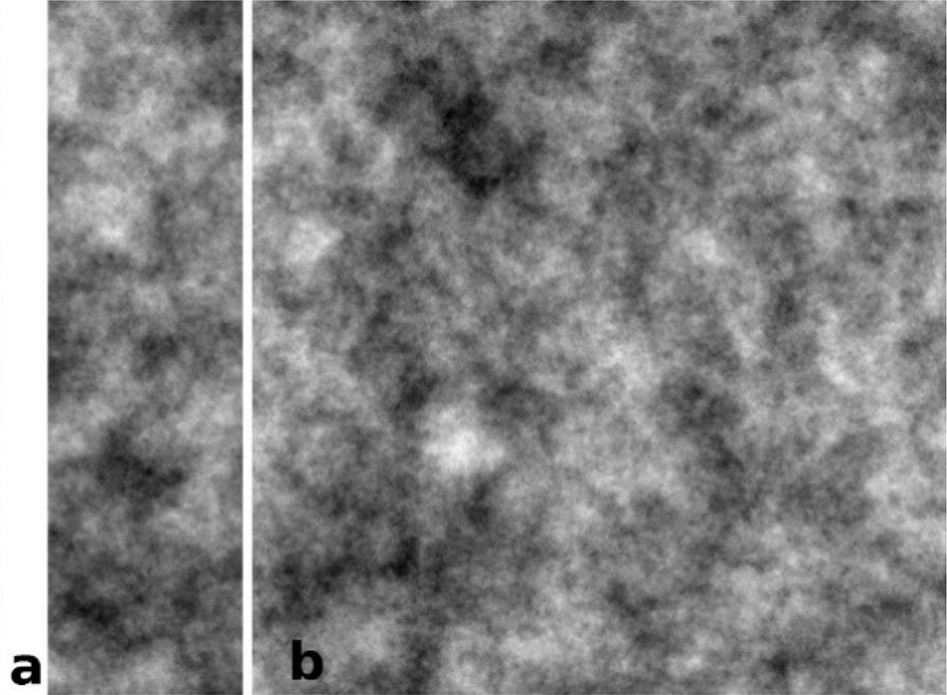
FS images of 8-bit grayscale with fractal dimension of 2.5. (**a**) 281 × 1000 pixels and (**b**) 1000 × 1000 pixels.

Because the icons to be analyzed had image resolutions between 281 × 1000 and 1000 × 1000 pixels, two images were generated with the same fractal dimension of 2.5 and with resolutions of 281 × 1000 and 1000 × 1000 pixels (Fig. 11).

**Figure 12:**
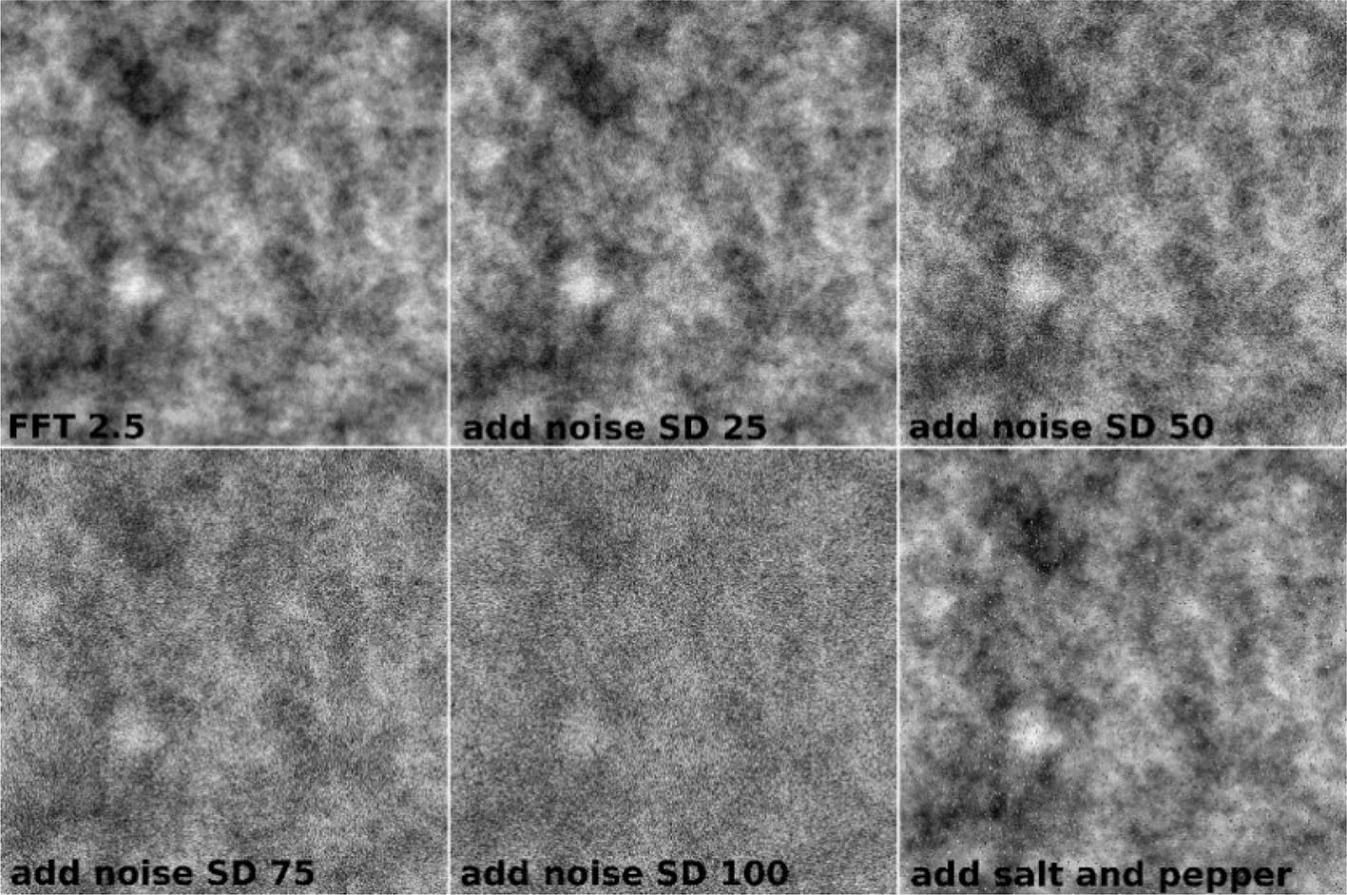
FS images of 8-bit grayscale with FD = 2.5. Added Gaussian noise with different SD and salt and pepper noise, at a resolution of 1000 × 1000 pixels.

Subsequently, these two images were altered by adding Gaussian noise with standard deviation (SD) 25, 50, 75, and 100 or by adding salt and pepper noise to test the ability of fractal or entropy analyses not robust against noise (Fig. 12). We used Gaussian noise and salt-and-pepper noise additively, since these two types of noise are the most common. In painting images, the multiplicative noise is not applicable, as it refers to an unwanted random signal that gets multiplied into some relevant signal during capture, transmission, or other processing, e.g. radar imagery. That is, it depends on the status of the system. For additive each pixel in the noisy image is the sum of the true pixel value and a random, Gaussian distributed noise value. Therefore, from the above rationale these noises were chosen. A more detailed investigation of different types of noise would be interesting but is far beyond the scope of this paper. Such a test was necessary because some icons were degraded and exhibit noise in their structure.

**Figure 13:**
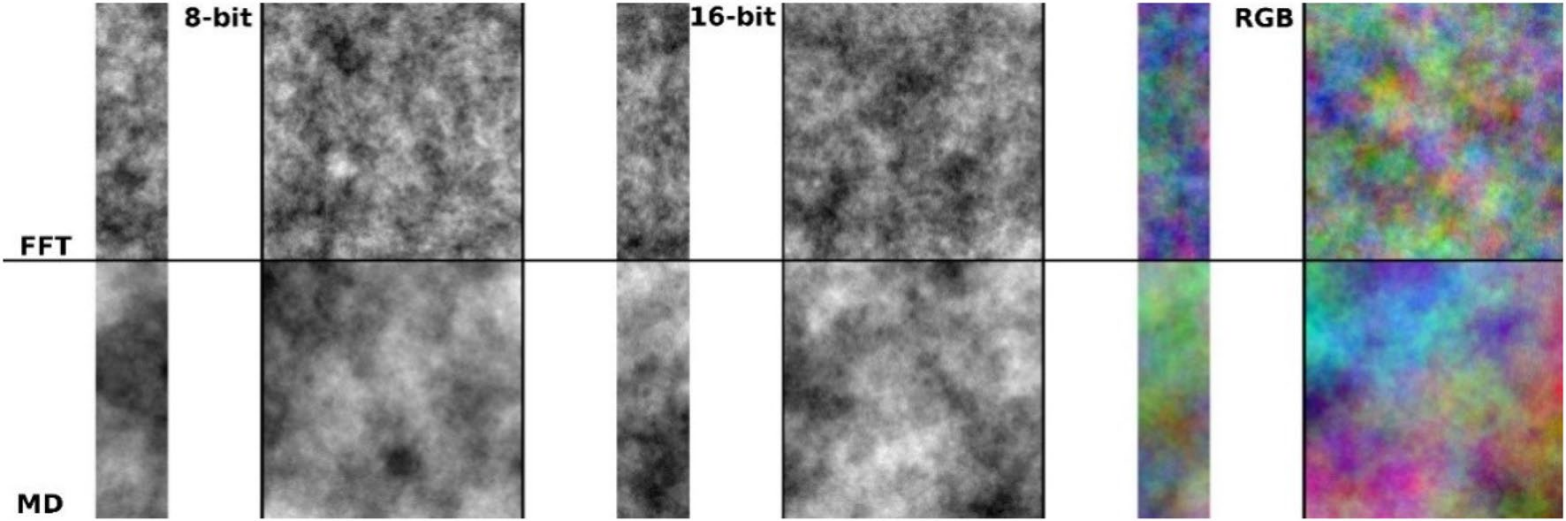
8-bit, 16-bit and RGB FS images with fractal dimension of 2.5. Upper panel: Inverse FFT algorithm. Lower panel: MD algorithm. Resolutions: 281 × 1000 pixels and 1000 × 1000 pixels.

To test the ability of KC to quantifying patterns for16-bit grayscale or RGB color images, two images with a resolution such as the smallest and largest icon (281 × 1000 and 1000 × 1000 pixels, respectively) were generated, using the Inverse FFT and the Midpoint Displacement (MD) algorithms (Fig. 13).

### Image acquisition and preprocessing

Icons were photographed with a digital camera (Nikon D60 digital camera, 18/55 VR lens, RAW format) mounted on a camera tripod (ZOMEI 55" Compact Light Weight Travel Portable Folding SLR Camera Tripod). Mostly original icons were photographed. A small number was taken from the electronic archives available on the Internet of some museums, collections or specialty albums. The details of the acquisition of the icon image sources are in the supplementary material. The RGB color images were converted to 8-bit grayscale using open-source software ImageJ 1.53^32^, and the resulting images were normalized using open-source software IQM 3.5—Histogram Modification—Normalization, with low and high offset 0.00%^19^. The conversion of the images to 8-bitgrayscale was necessary in order to be able to compare the proposed approach with the established methods of fractal and non-fractal analyses of image texture which rely on 8-bit images.

### Kolmogorov complexity

Kolmogorov complexity (KC) is part of the algorithmic information theory and is defined by the length in Bytes of the shortest software code to produce a result or an object^20^. This definition is a theoretical concept that cannot be solved in an exact way. It is not decidable if the actual version of the program is really the accurate one, thus estimates of the KC must be implemented, particularly for digital images. Writing a program to generate an object in a digital image might be feasible in some cases, but generally, it is simply not possible to do this. However, a reasonable approach is to take the amount of memory needed for a lossless compressed image as an estimate for the KC of that image^33^. It should be noted that compression is not a new trend and is affiliated to the entropy, e.g. the greater the Shannon entropy, say in bits, then the more random it looks. In contrast, the KC is the algorithmic complexity meant to quantify the algorithmic randomness. When an object cannot be compressed beyond its uncompressed length, the object is algorithmically random^34^.

Lossless compression algorithms are well investigated and optimized for digital images, e. g. PNG lossless image compression or ZIP compression of any file type. We opted in our analysis to use the PNG algorithm. PNG compression was chosen because the details of the algorithm are well known and corresponding implementations are very fast in the analysis and are freely available. We would like to point out that Zenil^34^ has presented the calculation of Kolmogorov complexity with the coding theorem method CTM and the block decomposition method BDM^34^. These two algorithms offer the advantage that the  Kolmogorov complexity can be computed more accurately, but unfortunately the technical effort is significantely higher. For this empirical study, the estimation via PNG compression turned out to be sufficient.

The 1200 icons, not having a close shape, yielded digital images at different resolutions: the smallest image had a resolution of 281 × 1000, and the largest 1000 × 1000. Unfortunately, the actual resolution affects the Kolmogorov complexity. Thus, with the same complexity, low resolution icons have a lower KC value than high resolution icons. Bringing the images to the same resolution could only be done by scaling an image in length or width, or by cropping. However, that would distort the image, and thus produce even greater bias in quantifying KC, becoming useless in the classification of patterns in the end. However, this bias was successfully eliminated by normalization, i.e. the ratio of KC to the image size in Mb, according to Eq. (1).

1 $$KC=\frac{{KC}_X}{S}$$

where *KC* is the Normalized Kolmogorov Complexity, ${KC}_X$ is Kolmogorov Complexity un-normalized (in MB) and *S* is image size (in MB).

Without normalization KC would present very large differences between resolutions (Fig. 1a,b). Normalization solves this impediment. There are still differences in resolution, but analyzed icons do not differ much in resolution. Thus, as can be seen in Fig. 2 of the manuscript, KC presents very close values for images at different resolution 281 × 1000 versus 1000 × 1000 pixels which makes us consider that for our sets of icons KC can be suitable in quantifying visual complexity.

Normalization KC allowed the quantification of complexity with values between 0 and 1, regardless of the image size. The value 0 is recorded when the image is uncomplicated, all pixels have the same gray value, and 1 is reached when each pixel is surrounded by the 8 neighbors with different gray values. As the value increases, so does the pixel inhomogeneity.

The relevance of the method used was tested by comparing the results obtained with those obtained by nine well-known fractal and non-fractal algorithms, using IQM 3.5: Logical depth, Differential Box-Counting, Relative Differential Box-Counting, Pyramid Dimension, Minkowski Dimension, FFT Dimension, Higuchi Dimension 1D, Higuchi Dimension 2D and Entropy^35–40^. KC proved to be the most relevant and fastest of the algorithms used. The comparative analysis is presented in the supplementary material.

## Supplementary Information

Supplementary Information.

## References

[1] Sigaki HYD, Perc M, Ribeiro HV (2018). History of art paintings through the lens of entropy and complexity. Proc. Natl. Acad. Sci. USA.

[2] Mureika JR, Fairbanks MS, Taylor RP, Stork DG, Coddington J, Bentkowska-Kafel A (2010). Multifractal comparison of the painting techniques of adults and children. Computer Vision and Image Analysis of Art.

[3] Shamir L (2015). What makes a Pollock: A machine vision approach. Int. J. Arts Technol..

[4] de la Calleja EM, Zenit R (2017). Topological invariants can be used to quantify complexity in abstract paintings. Knowl.-Based Syst..

[5] Kim D, Son SW, Jeong H (2014). Large-scale quantitative analysis of painting arts. Sci. Rep..

[6] Mandelbrot BB (1982). Fractal Geometry of Nature.

[7] Taylor RP, Micolich AP, Jonas D (1999). Fractal analysis of Pollock’s drip paintings. Nature.

[8] Taylor RP, Micolich AP, Jonas D (2002). The construction of Jackson Pollock’s fractal drip paintings. Leonardo.

[9] Oancea AV, Rapa A (2015). Some remarks on fractal analysis of Pollock’s paintings. Eur. J. Sci. Theol..

[10] Milovanović M, Tomić BM (2016). Fractality and self-organization in the orthodox iconography. Complexity.

[11] Forsythe A, Nadal M, Sheehy N, Cela-Conde CJ, Sawey M (2011). Predicting beauty: Fractal dimension and visual complexity in art. Br. J. Psychol..

[12] Hutter M (2004). Universal Artificial Intelligence: Sequential Decisions Based on Algorithmic Probability.

[13] Pacioli L (1509). De divina proportione.

[14] Henderson LD (2013). The Fourth Dimension and Non-Euclidean Geometry in Modern Art.

[15] Schattschneider DMC (2004). Escher: Visions of Symmetry.

[16] Ornes S (2019). Math Art: Truth, Beauty, and Equations.

[17] Bratitsi M, Liritzis I, Alexopoulou A, Makris D (2019). Visualising underpainted layers via spectroscopic techniques: A brief review of case studies. Sci. Cult..

[18] Bountis T, Fokas AS, Psarakis EZ (2017). Fractal analysis of tree paintings by Piet Mondrian (1872–1944). Int. J. Arts Technol..

[19] Kainz P, Mayrhofer-Reinhartshuber M, Ahammer H (2015). IQM: An extensible and portable open source application for image and signal analysis in Java. PLoS ONE.

[20] Kolmogorov A (1963). On tables of random numbers. Sankhyā Indian J. Stat. Ser. A.

[21] Alvarez-Ramirez J, Ibarra-Valdez C, Rodriguez E (2016). Fractal analysis of Jackson Pollock’s painting evolution. Chaos Solitons Fractals.

[22] Gruia KA (2019). The use of Sholl and Kolmogorov complexity analysis in researching on the sustainable development of creative economies in the development Region of Bucharest-Ilfov, Romania. Sustainability.

[23] Bratitsi M, Liritzis I, Vafiadou A, Xanthopoulou V, Palamara E, Iliopoulos Y, Zacharias N (2018). Critical assessment of chromatic index in archaeological ceramics by Munsell and RGB: Novel contribution to characterization and provenance studies. Mediter. Archaeol. Archaeom..

[24] Mureika JR, Dyer CC, Cupchik GC (2005). Multifractal structure in nonrepresentational art. Phys. Rev. E Stat. Nonlinear Soft Matter Phys..

[25] Sendler E (2005). The Icon, the Face of the Unseen.

[26] Pintilii R-D (2016). Determining forest fund evolution by fractal analysis (Suceava—Romania). Urban. Archit. Constr..

[27] Pintilii R-D (2017). Using fractal analysis in modeling the dynamics of forest areas and economic impact assessment: Maramureș County, Romania, as a case study. Forests.

[28] Andronache IC (2016). Fractal analysis for studying the evolution of forests. Chaos Solitons Fractals.

[29] Andronache I (2017). Assessment of textural differentiations in forest resources in Romania using fractal analysis. Forests.

[30] Andronache I (2019). Dynamics of forest fragmentation and connectivity using particle and fractal analysis. Sci. Rep..

[31] Daniel P (2020). Application of fractal–structural methods in the analysis of spatial distribution of the turnover in Romania. Econom. Comput. Econom. Cybernet. Stud. Res..

[32] Schneider CA, Rasband WS, Eliceiri KW (2012). NIH Image to ImageJ: 25 years of image analysis. Nat. Methods.

[33] Zenil H, Delahaye JP, Gaucherel C (2012). Image characterization and classification by physical complexity. Complexity.

[34] Zenil H (2020). A review of methods for estimating algorithmic complexity: Options, challenges, and new directions. Entropy.

[35] Haralick RM, Shanmugam K, Dinstein I (1973). Textural features for image classification. IEEE Trans. Syst. Man Cybern..

[36] Marana, A. N., Costa, L. da F., Lotufo, R. A. & Velastin, S. A. Estimating crowd density with Minkowski fractal dimension. In *ICASSP, IEEE International Conference on Acoustics, Speech and Signal Processing—Proceedings* **6**, 3521–3524 (1999).

[37] Ahammer H (2011). Higuchi dimension of digital images. PLoS ONE.

[38] Spasić S (2014). On 2D generalization of Higuchi’s fractal dimension. Chaos Solitons Fractals.

[39] Kesić S, Spasić SZ (2016). Application of Higuchi’s fractal dimension from basic to clinical neurophysiology: A review. Comput. Methods Programs Biomed..

[40] Mayrhofer-Reinhartshuber M, Ahammer H (2016). Pyramidal fractal dimension for high resolution images. Chaos Interdiscip. J. Nonlinear Sci..

