# Supplementary material for: Kolmogorov compression complexity may differentiate different schools of Orthodox iconography
Source: Sci Rep. 2022 Jun 24;12:10743. doi: 10.1038/s41598-022-12826-w (PMC9232591; doi:10.1038/s41598-022-12826-w)
Supplement: Supplementary file 1 — Supplementary Information. [file 41598_2022_12826_MOESM1_ESM.docx]

**Kolmogorov compression complexity may differentiate different schools of Orthodox iconography**

Daniel Peptenatu^1^_,_ Ion Andronache^1,^*, Helmut Ahammer^2^, Richard Taylor^3^, Ioannis Liritzis**^4^,** Marko Radulovic^5^, Bogdan Ciobanu^6,7^, Marin Burcea^8^, Matjaz Perc^9,10,11,12^, Tuan D. Pham^13^, Bojan M. Tomić^14^, Cosmin Iulian Cirstea^15^, Adrian Lemeni^15^, Andreea Karina Gruia^1,8^, Alexandra Grecu^1,8^, Marian Marin^1^, Herbert F. Jelinek^16^

^1^Research Center for Integrated Analysis and Territorial Management, Faculty of Geography, University of Bucharest, Bucharest 030018, Romania;

^2^GSRC, Division of Biophysics, Medical University of Graz, Graz 8010, Austria;

^3^Physics Department, University of Oregon, Eugene, OR 97403, USA;

^4^**Key Research Institute of Yellow River Civilization and Sustainable Development & Collaborative Center on Yellow River Civilization, Laboratory of Yellow River Cultural Heritage, Henan University, Kaifeng 475001, Henan, China;**

^5^ Laboratory of Cancer Cell Biology, Institute for Oncology and Radiology, University of Belgrade, Belgrade, 11000, Serbia;

^6^Mural Art Department, Faculty of Decorative Arts and Design, Bucharest National University of Arts, Bucharest, Romania;

^7^Union of Visual Artists in Romania, Bucharest, Romania;

^8^Faculty of Administration and Business, University of Bucharest, Bucharest, Romania;

^9^Faculty of Natural Sciences and Mathematics, University of Maribor, Koroška cesta 160, 2000 Maribor, Slovenia;

^10^Department of Medical Research, China Medical University Hospital, China Medical University, Taichung 404332, Taiwan;

^11^Alma Mater Europaea, Slovenska ulica 17, 2000 Maribor, Slovenia;

^12^Complexity Science Hub Vienna, Josefstädterstraße 39, 1080 Vienna, Austria;

^13^Center for Artificial Intelligence, Prince Mohammad Bin Fahd University, Khobar, 31952, Saudi Arabia;

^14^Institute for Multidisciplinary Research, University of Belgrade, 1 Kneza Višeslava st., 11030 Belgrade, Serbia.

^15”^Dumitru Stăniloae” Doctoral School, Faculty of Orthodox Theology, University of Bucharest;

^16^Department of Biomedical Engineering and Health Engineering Innovation Center, Khalifa University, Abu Dhabi, United Arab Emirates

/*-ș/țî

*Corresponding author: Ion Andronache; Tel.: +40757722170 E-mail: [ion.andronache@geo.unibuc.ro](mailto:ion.andronache@geo.unibuc.ro)

**Supplementary material**

To highlight the relevance of the proposed analysis of the three sets of icons, eight fractal parameters and one non-fractal parameter were analyzed (logical depth, differential Box-Counting, relative differential Box-Counting, Pyramid Dimension, Minkowski Dimension, FFT Dimension, Higuchi Dimension 1D and 2D, and entropy). The results show the usefulness of Kolmogorov Complexity KC in identifying the differences between the three schools of iconographic painting (Analytical spreadsheet data accompanied by fractal surfaces generated images and icons from five case studies - one case for a validation of Kolmogorov Complexity normalization and 4 cases to validate the analysis of the other 1200 icons- as well as IQM software are given in https://drive.google.com/drive/folders/1CpGUUufz40DDYUR6uFa5CRzC9lEJDNpk?usp=sharing).

**Methodology**

The following fractal algorithms were analyzed:

**1. Logical depth (LD).** Logical depth is another concept of the algorithmic information theory and is defined by the time it takes to run that shortest software program^1^. Using computers for this measurement, it is necessary to run the program several times and determine averages. Computers asynchronously process a huge number of tasks and a delay for a specific task is always feasible. Actual computations of Kolmogorov complexities and logical depths were performed by using the image processing software IQM. Lossless PNG compression was chosen for estimating Kolmogorov complexities. In addition, we also present image size normalized KC values. Averages for logical depths were computed with n=10.

**2. Differential Box-Counting (Db_DBC).** The common Box-Counting algorithm uses boxes with distinct scales and counting if subparts of the objects under investigation are located therein. It is usually applied to binary images rather than grey value images.

Differential Box-Counting is an extension of this algorithm, where cubes rather than boxes are used. The third dimension of these cubes is the grey value of an image and thus, grey value images can be investigated^2^.

**3. Relative Differential Box-Counting (Db_RDBC).** The Relative Differential Box-Counting algorithm further extends the Differential Box-Counting algorithm by computing relative grey value differences at different scales rather than differences between the minimum and maximum grey levels^3^.

**4. Pyramid Dimension Gradient - PGM method (Dp_Gradient (PGM).** Pyramid Dimension is a fractal analysis derived from Box-Counting that uses pyramid images rather than boxes. These pyramids are image sequences at different sizes^2^. The more fragmented and uneven the distribution of gray tones is, the larger is the Pyramid Dimension and vice versa. The analysis was performed using IQM 3,5- Pyramid Gradient Method (PGM), according to Mayrhofer-Reinhartshuber and Ahammer^2^.

**5. Minkowski Dimension - Blanket method (Dm_Blanket).** Minkowski Dimension analyzes the relationship between the object and the space occupied by it and is obtained by calculating the volume of the gray value expanded object. The higher the non-uniformity of the fractal objects are, the higher is the Minkowski fractal dimension and vice versa. The analysis was performed using IQM 3.5 - Pyramid Gradient Method (PGM), according to Marana *et al.*^3^.

**6. Fractal FFT Dimension using Discrete Fourier Transformation (Df).** This is a method of characterizing fractal surfaces using frequency analysis. The higher the complexity of the image, the higher is the FFT fractal dimension and vice versa. The analysis was performed using IQM 3.5, according to Mayrhofer-Reinhartshuber and Ahammer^2^.

**7. Higuchi Dimension 1D (Dh).** The Higuchi Dimension 1D (H1D) method is appropriate to measure textural features and allows the quantification of the degree of complexity of the analysed object, especially when the images are anisotropic. Each row and column of an image (representing the intersection of the gray value of the surface with the 2D plane of the image) was extracted from the grayscale representation, and then analyzed separately. The analysis was performed using IQM 3.5, according to Ahammer^5^.

**8. Higuchi 2D - KfoldDiff method (Dh-KfoldDiff).** Higuchi Dimension 2D (H2D) analysis is based on the calculation of the fractal complexity of an image, considering the relation of the gray tones of some pixels to the gray tones of the pixels in their vicinity. H2D is a generalization of H1D for calculating the fractal dimension of a surface^6^. The analysis was performed using IQM 3.5, KfoldDiff method, according to Ahammer *et al.*^7^.

**9. Entropy.** Entropy is a statistical measure of the disorder or complexity of an image. Entropy is high if the image is not texturally uniform and many elements of the gray-level co-occurrence matrix have very small values. Thus, complex textures tend to have high entropy. The higher the entropy value, the higher the textural disorder. When the entropy is zero it means that there is no disorder. Entropy was calculated with IQM 4.0 according to Haralick *et al.*^8^.

**Results**

**Fractal dimensions of FS image sets in 8-bit grayscale.** The 11 sets (each set having 20 images) of Inverse FFT generated images in 8-bit grayscale and with fractal dimensions from 2.0 to 3.0 (as FD=2.0, 2.1…3.0) were analyzed with all eight fractal parameters and with entropy using IQM 3.5 software. The painting on canvas is made by pigments and a paintbrush, thus produce an oftentimes perceptible relief. This together with the geometrical image provide 2<FD<3. Yet in a 2D image the FD can be even < 1; but since in our case we allow unlimited resolution, and everything is made of pixels, which have fractal dimension 2, the chosen 2<FD<3 is justified.

The aim for these images, is to identify the best parameter identifying complexity i.e. images in one set have dissimilar values than images in another set which in turn is useful in the identification of the patterns (Figure S1).

| 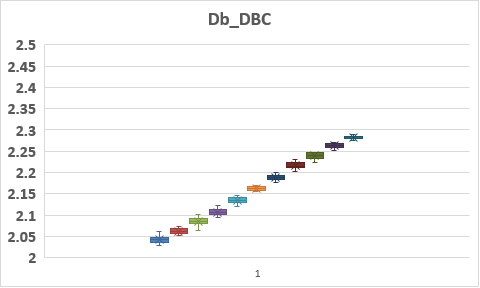 | 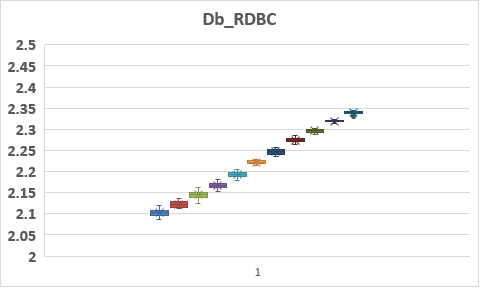 |
| --- | --- |
| 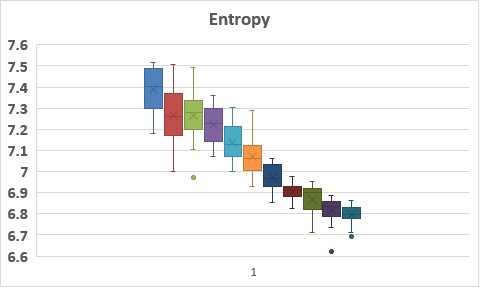 | 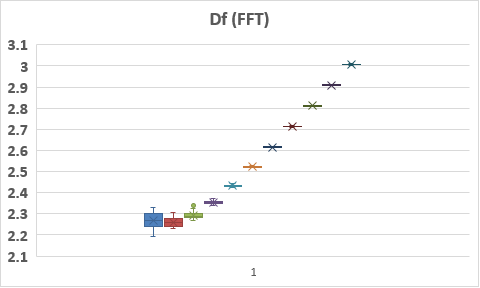 |
| 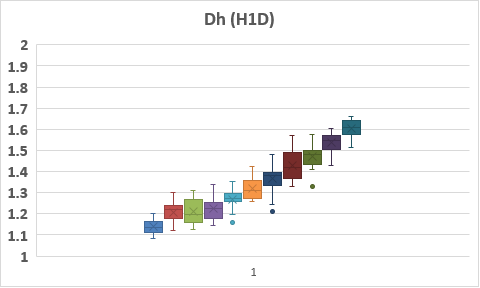 | 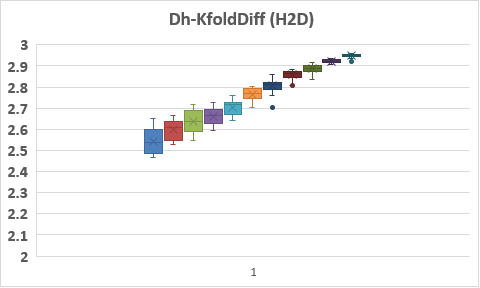 |
| 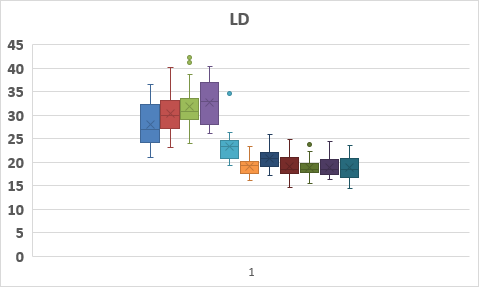 | 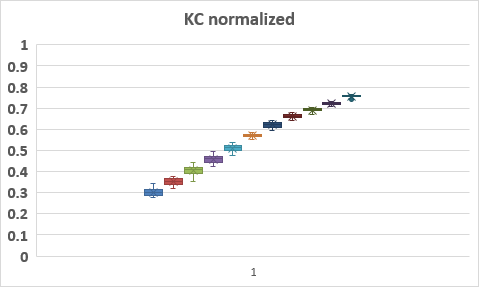 |
| 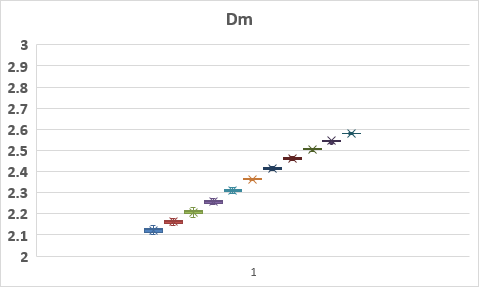 | 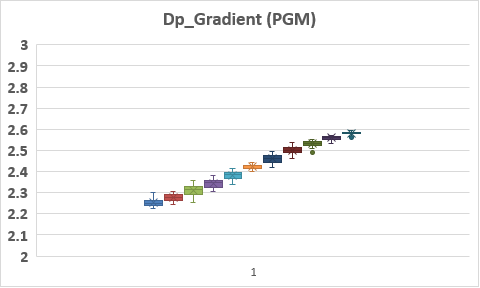 |
| **Figure S1**. Box-plots of the sets of FS images generated at 1024x1024 resolution. The best pattern differentiations were obtained with Kolmogorov Complexity (KC normalized) and the weakest differentiation were obtained with Logical Depth (LD) and Entropy. | |

From the analyses performed, the best pattern differentiations were obtained with Minkowski Dimension, Differential Box-Counting, Relative Differential Box-Counting, Kolmogorov Complexity (KC normalized), but also partially with Pyramid Dimension and FFT Dimension (Fig. S1). Not so good were Higuchi Dimension 1D and 2D, Logical Depth, but also Entropy. The second category of images generated with the Inverse FFT algorithm and fractal dimension of 2.5 had values such as for FD=2.4 or FD=2.6. Therefore, their ability to identify the class they come from is reduced.

1. **Fractal analysis of FS images in 8-bit grayscale at 281x1000 and 1000x1000 pixels resolution.** In the second stage, the identification of the parameter which best describes the complexity we generated 2 FS images in grayscale 281x1000 and 1000x1000 pixels resolution is achieved with the Inverse FFT algorithm at similar resolutions with the smallest icon versus the largest icon. If the difference between the fractal size and/or the entropy of the small image is very close to that of the large image, it means that that parameter is not sensitive to the image size and therefore can be useful in comparing images with different sizes.


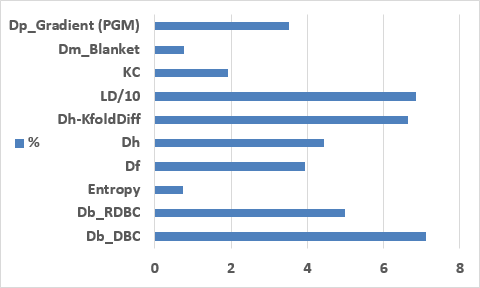


**Figure S2.** The ratio between the two images with low 281x1000 and high 1000x1000 pixels resolution generated with Inverse FFT algorithm, for the 10 parameters of fractal dimension and entropy values.

Figure S2 shows that Entropy, Minkowski Dimension and Kolmogorov Complexity offer the smallest differences, being the least sensitive to image size changes.

Although entropy offers the best solution here, it is not suitable when analyzing images with close fractal dimensions.

**3. Fractal analysis of FS images in 8-bit grayscale at 281x1000 and 1000x1000 pixels resolution affected by Gaussian noise or Salt and Pepper noise.** Fractal and entropy analysis on FS images, distorted with Gaussian noise or Salt and Pepper noise, showed that the best measures are Kolmogorov Complexity, Minkowski Dimension and Entropy. These three measures showed the least dependency on noise.


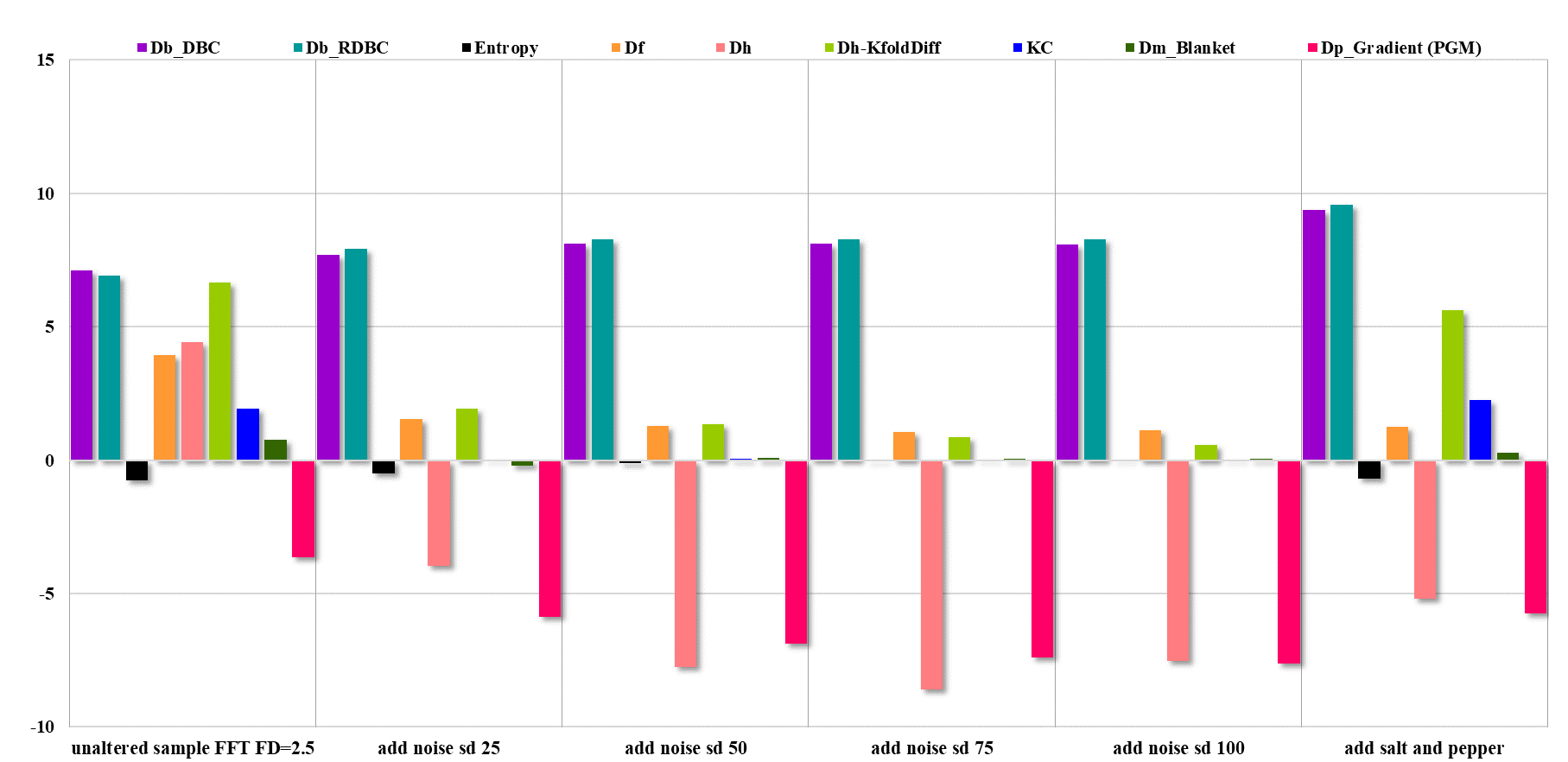


**Figure S3.** The ratio between fractal and entropy measures for the two images with a resolution 1000x1000 pixels, non-altered and altered with Gaussian noise and Salt and Pepper noise. The ratio between the values of fractal and entropy measures for the two images with a resolution of 281x1000 pixels and 1000x1000 pixels, altered with Gaussian noise and Salt and Pepper noise. Colored bars denote the greater / smaller fractal or entropy dimension as noise effect per fractal algorithm.

In Figure S3 it can be clearly seen that the images with and without noise have almost identical KC values, regardless of whether they have a resolution of 281x1000 or 1000x1000. For all of the cases of modified noise levels, the biggest quantification differences were Differential Box-Counting, Relative Differential Box-Counting, Higuchi 1D Dimension and Pyramid Dimension. The smallest were the differences of Kolmogorov Complexity, FFT Dimension, Higuchi 2D and Entropy. For example, the negative values for Higuchi 1D and Pyramid Dimension PGM, are as such because the FD of the noise image is lower than that of the original images. For the rest the FD of the image with noise is higher than the FD of the original images, without noise added (same applies for Entropy).

As the images of our icons show artifacts imposed by the passage of several centuries, like noise, we chose Kolmogorov Complexity as the main method for the analysis of icons. Hence, after the noise alteration of the analyzed images, the KC value is only slightly modified.

**Normalized KC analysis of the three sets of Pollock paintings.** The classical fractal dimension (box counting fractal dimension, BCFD) of Jackson Pollock’s painting used by Alvarez-Ramirez et al., (2016)^22^, were compared to our KC results. In order to validate KC nine paintings by Pollock (Figure S4) from the 3 stages of his creative activity, were analysed: early (a. Figures in a landscape – 1937, b. The flame – 1938, c. Birth - 1941), drip (d. Number 17 – 1949, e. Mural on Indian red ground – 1950, f. Number 1 -Lavender mist - 1950) and late (g. Untitled 2 - 1951, h. Blue poles Number 11- 1952, i. Ocean greyness - 1953) periods. The results (Figure S5) show that normalized KC increases from the early stage to the drip stage and towards the end of his career it decreases slightly. The results obtained with KC confirm the results of previous research thus KC can quantify visual complexity.


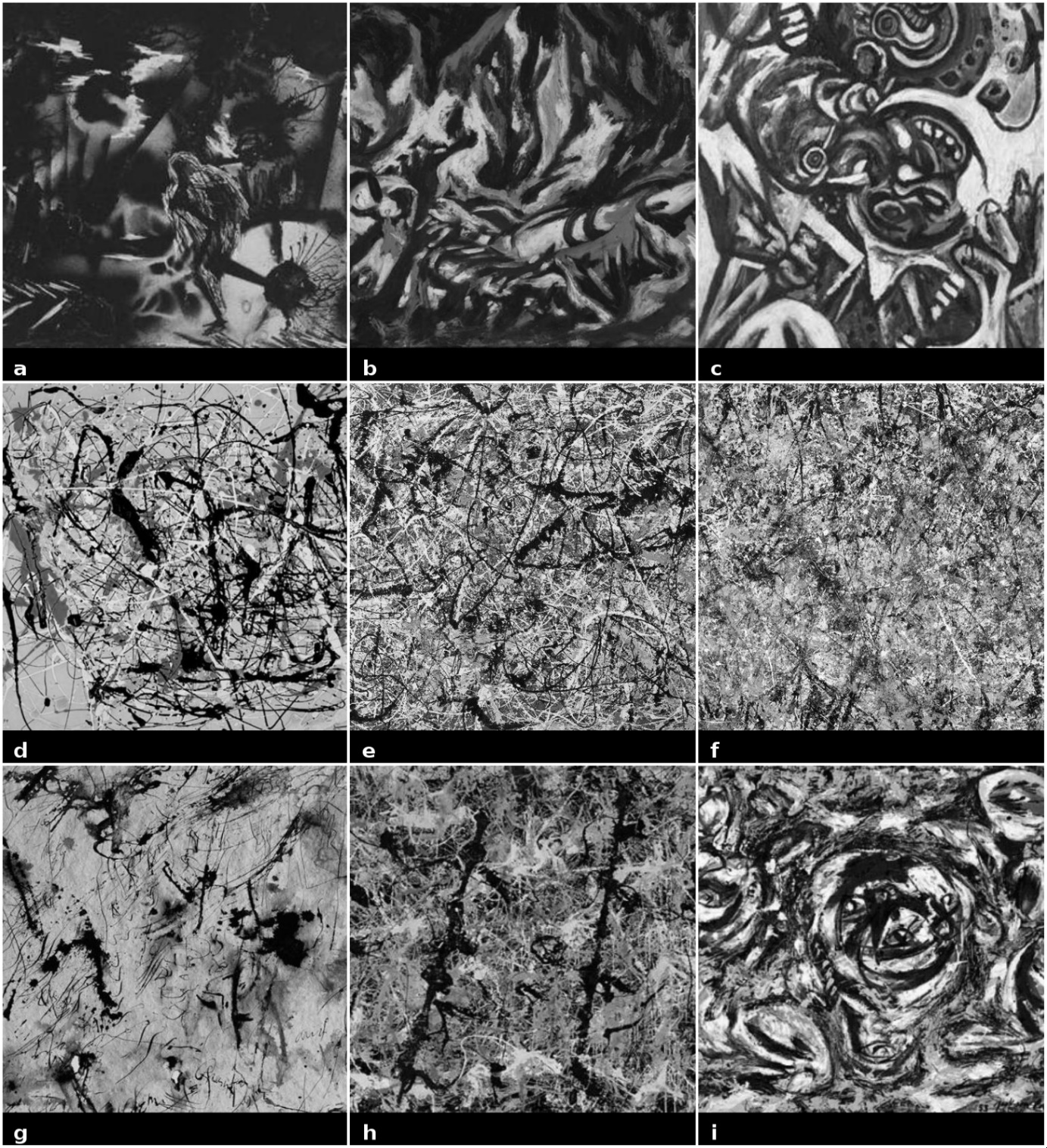

**Figure S4.** Pollock's paintings from his 3 stages of activity: early stage (a. Figures in a landscape – 1937, b. The flame – 1938, c. Birth - 1941); drip stage (d. Number 17 – 1949, e. Mural on Indian red ground – 1950, f. Number 1 -Lavender mist - 1950) and late stage (g. Untitled 2 - 1951, h. Blue poles Number 11- 1952, i. Ocean greyness - 1953). The paintings have been converted to gray-scale.


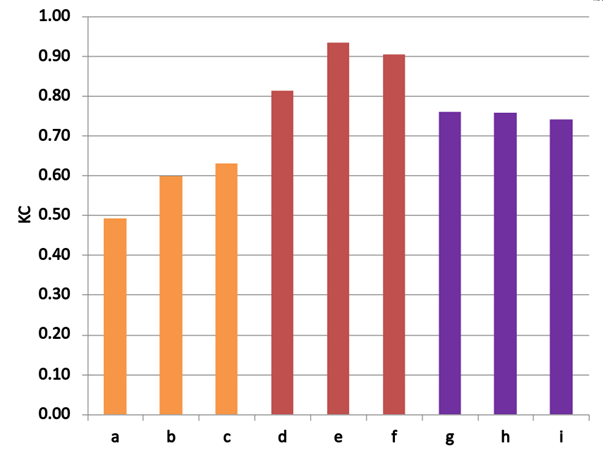
**Figure S5.** Normalized KC of Pollock's paintings from the 3 stages of his creative activity: a, b, c - early stage; d, e, f - drip stage and g, h, i late stage. It can be seen that the degree of visual complexity increases from early stage to drip stage, the stage that established him as a very important painter and decreases slightly in late stage.

**Image acquisition**

Icons were photographed with a digital camera (Nikon D60 digital camera, 18/55 VR lens, RAW format) mounted on a camera tripod (ZOMEI 55" Compact Light Weight Travel Portable Folding SLR Camera Tripod). Mostly original icons were photographed. A small part were taken from

1. the electronic archives available on the Internet of some museums:
   - Bistrița-Neamț Monastery and Museum (<https://bistrita.mmb.ro/>)
   - Văratec Museum (<http://www.manastireavaratic.ro/muzeu/>)
   - Văleni Parish Piatra Neamț (<https://valenipiatraneamt.mmb.ro/>)
   - Buzău Episcopate Museum (<https://arhiepiscopiabzvn.ro/muzeul-bisericesc-eparhial.html>)
   - Curtea de Argeș Monastery (<https://www.eparhiaargesului.ro/mrea_argesului.html>)
   - Agapia Nouă Monastery (<https://agapia.mmb.ro/>)
   - Sfântul Gheorghe Church from Pitești (<http://www.bisericadomneascapitesti.ro/>)
   - Pângărați Monastery (<https://pangarati.mmb.ro/>)
   - Sucevița Monastery (<https://manastirea-sucevita.ro/>)
   - Iași Metropolitan Palace (<https://ansamblulmitropolitaniasi.ro/resedinta-mitropolitana/palatul-mitropolitan>)
   - Secu Monastery Museum, (<https://secu.mmb.ro/muzeul-manastirii-secu>)
   - Roman Episcopate Museum (<https://eprb.ro/muzeu>)
   - Maramureș Museum from Sighetul Marmației (<https://muzeulmaramuresului.ro/>)
   - Museum of the Romanian Patriarchate - Antim Monastery (<http://manastireaantim.ro/muzeul/>)
   - Voroneț Monastery (<https://www.manastireavoronet.ro/>)
   - Cozia Monastery (http://cozia.arhiepiscopiaramnicului.ro/)
2. For Figure S4: the images were taken as follows:
   - early stage (a. Figures in a landscape – 1937 - https://www.moma.org/collection/works/61658) b. The flame – 1938, https://www.moma.org/collection/works/79680, c. Birth – 1941 https://www.tate.org.uk/art/artworks/pollock-birth-t03979; https://www.wikiart.org/en/jackson-pollock/birth);
   - drip stage (d. Number 17 – 1949 https://www.wikiart.org/en/jackson-pollock/number-17-1949 , e. Mural on Indian red ground – 1950, and f. Number 1 - Lavender mist – 1950 https://www.mcgawgraphics.com/products/jackson-pollock-number-1-1950-lavender-mist-1950)
   - late stage (g. Untitled 2 - 1951,https://www.moma.org/collection/works/34857 , h. Blue poles Number 11- 1952 https://en.wikipedia.org/wiki/Blue_Poles, and i. Ocean greyness – 1953 https://www.guggenheim.org/artwork/3486). These images are under the Creative Commons Attribution-ShareAlike License 3.0 and have been converted to gray-scale for analysis.
3. Collections and specialty albums^10-21^

All images are under the Creative Commons Attribution-ShareAlike License 3.0.

**REFERENCES**

1. Bennett, C. H. Logical Depth and Physical Complexity. In A half-century survey on The Universal Turing Machine, 207–235, doi:10.1007/978-3-7091-6597-3_8 (1995).
2. Sarkar, N., und B. B. Chaudhuri. An Efficient Approach to Estimate Fractal Dimension of Textural Images. Pattern Recognition 25, Nr. 9 (September 1992): 1035–41.
3. Jin, X. C., S. H. Ong, und Jayasooriah. A practical method for estimating fractal dimension. *Pattern Recognition Letters* 16, Nr. 5 (Mai 1995): 457–64. https://doi.org/10.1016/0167-8655(94)00119-N.
4. Mayrhofer-Reinhartshuber, M. & Ahammer, H. Pyramidal fractal dimension for high resolution images. *Chaos: An Interdisciplinary Journal of Nonlinear Science* 26, 073109, https://doi.org/10.1063/1.4958709 (2016).
5. Marana, A. N., Costa, L. da F., Lotufo, R. A. & Velastin, S. A. Estimating crowd density with Minkowski fractal dimension. ICASSP, IEEE International Conference on Acoustics, Speech and Signal Processing - Proceedings 6, 3521–3524, DOI: 10.1109/ICASSP.1999.757602 (1999).
6. Ahammer, H. Higuchi Dimension of Digital Images. *PLoS ONE* 6, 9, e24796. https://doi.org/10.1371/journal.pone.0024796 (2011).
7. Spasić, S. On 2D generalization of Higuchi’s fractal dimension. *Chaos, Solitons and Fractals* 69, 179–187, https://doi.org/10.1016/j.chaos.2014.09.015 (2014).
8. Ahammer, H., Sabathiel, N. & Reiss, M. A. Is a two-dimensional generalization of the Higuchi algorithm really necessary? *Chaos* 25, 073104, https://doi.org/10.1063/1.4923030 (2015).
9. Haralick, R. M., Shanmugam, K. & Dinstein, I. Textural Features for Image Classification. IEEE Transactions on Systems, Man, and Cybernetics SMC-3, 6, 610-621, DOI: 10.1109/TSMC.1973.4309314 (1973).
10. *Arta din Moldova de la Ştefan cel Mare la Movileşti*, Ministerul Culturii, Muzeul Naţional de Artă al României, Secţia de artă medievală românească, Bucureşti, 1999.
11. Bakirtzis Charalampos, *Ayios Nikolaos Orphanos - The wall paintings*, Akritas Publications, 2003.
12. Chatzidakis Nano, *Arta Greacă: Icoanele bizantine*, Editura Ekdotike Athenon, Grecia, 1994.
13. Efremov, Alexandru, *Icoane românești*, ed. Meridiane, București, 2002.
14. Florenski, Pavel, *Iconostasul*, Fundaţia Anastasia, București, 1994.
15. Kazanaki-Lappa Maria, *Arte Bizantina e Postbizantina a Venezia –Museo di Icone dell'Istituto Elennico di Studi Bizantini e Postbizantini di Venezia*, Eurocrom Libri, 2009.
16. Kondakov Nikodim, *Icoane*, Editura Cartier, Chişinău, 2012.
17. Lăzărescu Anca, *Capodopere din Evul Mediu românesc*, Editura Alcor Edimpex, Bucureşti, 2006.
18. Panselinos Manouel, *Protato*, Editura sfintelor Mănăstiri ale Sfântului Munte Athos, Thesalonic, 1997.
19. Petkovic Sreten, Slavomir Matejic, *Icoanele Mănăstirii Chilandar*, Editura Monastery Chilandar, Sfântul Munte Athos, Grecia, 1997.
20. Simionovici, monahia Elena, *Icoane. Sfânta Mănăstire Voroneţ*, Editura Thausib, Sibiu, 2004.
21. Tsigaridas, Eftimios N., *The frescoes in the Parekklesion of St. Euthymios in the Basilica of St. Demetrios. The work of Manuel Panselinos in Thessaloniki*, P. Pournaras Editions, Thessaloniki, 2008.
22. Alvarez-Ramirez J, Ibarra-Valdez C, Rodriguez E. Fractal analysis of Jackson Pollock’s painting evolution. *Chaos Solitons Fractals* 83, 97–104, doi:10.1016/j.chaos.2015.11.034 (2016)
